# Modelling technical and biological biases in macroinvertebrate community assessment from bulk preservative using multiple metabarcoding markers

**DOI:** 10.1111/mec.15620

**Published:** 2020-10-12

**Authors:** Filipa M. S. Martins, Miguel Porto, Maria J. Feio, Bastian Egeter, Aurélie Bonin, Sónia R. Q. Serra, Pierre Taberlet, Pedro Beja

**Affiliations:** ^1^ Departamento de Biologia Faculdade de Ciências Universidade do Porto Porto Portugal; ^2^ CIBIO/InBio Centro de Investigação em Biodiversidade e Recursos Genéticos Universidade do Porto Vila do Conde Portugal; ^3^ CIBIO/InBio Centro de Investigação em Biodiversidade e Recursos Genéticos Instituto Superior de Agronomia Universidade de Lisboa Lisboa Portugal; ^4^ Departamento de Ciência da Vida Centro de Ciências do Mar e do Ambiente, MARE Universidade de Coimbra Coimbra Portugal; ^5^ Laboratoire d'Ecologie Alpine (LECA) CNRS Université Grenoble Alpes Grenoble France; ^6^ Tromsø Museum UiT – The Arctic University of Norway Tromsø Norway

**Keywords:** biomonitoring, detection probability, ecological indicators, high‐throughput sequencing, multimarker metabarcoding

## Abstract

DNA metabarcoding from the ethanol used to store macroinvertebrate bulk samples is a convenient methodological option in molecular biodiversity assessment and biomonitoring of aquatic ecosystems, as it preserves specimens and reduces problems associated with sample sorting. However, this method may be affected by errors and biases, which need to be thoroughly quantified before it can be mainstreamed into biomonitoring programmes. Here, we used 80 unsorted macroinvertebrate samples collected in Portugal under a Water Framework Directive monitoring programme, to compare community diversity and taxonomic composition metrics estimated through morphotaxonomy versus metabarcoding from storage ethanol using three markers (COI‐M19BR2, 16S‐Inse01 and 18S‐Euka02) and a multimarker approach. A preliminary in silico analysis showed that the three markers were adequate for the target taxa, with detection failures related primarily to the lack of adequate barcodes in public databases. Metabarcoding of ethanol samples retrieved far less taxa per site (alpha diversity) than morphotaxonomy, albeit with smaller differences for COI‐M19BR2 and the multimarker approach, while estimates of taxa turnover (beta diversity) among sites were similar across methods. Using generalized linear mixed models, we found that after controlling for differences in read coverage across samples, the probability of detection of a taxon was positively related to its proportional abundance, and negatively so to the presence of heavily sclerotized exoskeleton (e.g., Coleoptera). Overall, using our experimental protocol with different template dilutions, the COI marker showed the best performance, but we recommend the use of a multimarker approach to detect a wider range of taxa in freshwater macroinvertebrate samples. Further methodological development and optimization efforts are needed to reduce biases associated with body armouring and rarity in some macroinvertebrate taxa.

## 1 INTRODUCTION

Biological monitoring (i.e., biomonitoring) is a key element of integrated freshwater management (Jackson et al., 2016), which is mandatory under regulations such as the European Water Framework Directive (WFD; Directive 2000/60/EC) and the 1972 US Clean Water Act. Biomonitoring provides information on spatial and temporal trends in the ecological state of ecosystems, allowing recognition of current or forthcoming threats and enacting early corrective measures where needed (Jackson et al., 2016). To meet these goals, biomonitoring commonly requires a taxonomical characterization (e.g., species richness, composition, and relative or absolute abundances) of the communities of target organisms such diatoms, macrophytes, benthic macroinvertebrates or fish. These community parameters are then used to compute biological indices reflecting the degree of stressor impacts at sampling sites (Jackson et al., 2016; Pawlowski et al., 2018). Traditionally, such characterization involves expensive, laborious and time‐consuming morphological identification of organisms, which requires high taxonomic expertise, provides limited taxonomic resolution for early life stages, and strongly constrains the spatial and temporal coverage of sites that can be sampled (Baird & Hajibabaei, 2012; Leese et al., 2016). During the past decade, it was demonstrated that these constraints can at least partly be offset by molecular techniques such as DNA metabarcoding, which allows the identification of multiple taxa from environmental samples using standard genetic markers (Baird & Hajibabaei, 2012; Taberlet, Coissac, Pompanon, Brochmann, & Willerslev, 2012). Consequently, efforts have been undertaken to integrate metabarcoding in freshwater monitoring (Elbrecht & Steinke, 2019; Feio et al., 2020; Leese et al., 2016; Pawlowski et al., 2018), which for organisms such as diatoms have succeeded in producing methods that are virtually ready for practical application (Apothéloz‐Perret‐Gentil et al., 2017; Cordier, Lanzén, Apothéloz‐Perret‐Gentil, Stoeck, & Pawlowski, 2019; Mortágua et al., 2019; Visco et al., 2015), whereas for others, a great deal of optimization and standardization is still needed (Pawlowski et al., 2018).

Benthic macroinvertebrates are a group of organisms widely used in freshwater monitoring (Jackson et al., 2016), for which there are still uncertainties on the best molecular approaches to replace morphological methods (Blackman et al., 2019; Bush et al., 2019). One approach involves the metabarcoding of environmental DNA (eDNA) extracted from water samples, but recent studies suggest that this may be largely unsuitable to characterize local communities of macroinvertebrate indicator taxa (Macher et al., 2018; Pereira‐da‐Conceicoa et al., 2019). More frequently, the proposed approaches aim at replicating as closely as possible the traditional methods, and they involve collecting macroinvertebrate bulk samples in the field, separating individuals from stones, twigs, algae and other materials, grinding each sorted sample to produce a tissue homogenate, and DNA metabarcoding of a subsample of the homogenate (Elbrecht & Steinke, 2019). This approach has proved successful at retrieving the taxa identified morphologically (Elbrecht, Vamos, Meissner, Aroviita, & Leese, 2017; Emilson et al., 2017; Serrana, Miyake, Gamboa, & Watanabe, 2019), but (a) it involves the destruction of samples, which is incompatible with the sample preservation requirements of national regulatory agencies (e.g., under WFD), and (b) it maintains the sorting step that is time consuming and may increase the chances of cross‐contamination (Elbrecht, Peinert, & Leese, 2017). Sorting can potentially be reduced by grinding unsorted bulks after removing only the very coarse materials (Hajibabaei et al., 2019; Majaneva, Diserud, Eagle, Hajibabaei, & Ekrem, 2018), making the approach more expeditious, yet it does not avoid sample destruction. To offset these problems, metabarcoding can be performed using DNA extracted from the solution used to preserve the invertebrate samples, usually ethanol, as organisms release cells and free DNA into the preservative medium (Shokralla, Singer, & Hajibabaei, 2010). Studies have shown that DNA extracted from preservative solution can indeed detect the organisms present in mock communities (Carew, Coleman, & Hoffmann, 2018; Gauthier et al., 2020; Hajibabaei, Spall, Shokralla, & van Konynenburg, 2012; Nielsen, Gilbert, Pape, & Bohmann, 2019; Shokralla et al., 2010), with a few additional studies also showing promising results using coarse and unsorted field samples, that contain potential PCR inhibitors from river substrate and organic matter (Martins et al., 2019; Zizka, Leese, Peinert, & Geiger, 2019). However, further research is needed on potential limitations and shortcomings associated with this approach (Blackman et al., 2019; Bush et al., 2019).

Previous studies have underlined that metabarcoding of preservative solutions may have differential ability to detect different macroinvertebrate taxa, which may introduce biases in estimates of the taxonomic composition of bulk samples (reviewed in Table 1). Some of these biases are transversal to metabarcoding workflows and include (a) the lack of complete barcoding reference database, limiting sequence identification at lower taxonomic ranks in less represented groups (Elbrecht et al., 2017; Erdozain et al., 2019; Weigand et al., 2019), and (b) primer specificity causing some taxonomic groups to be rarely amplified or missed altogether under certain conditions (Carew et al., 2018; Elbrecht & Leese, 2017b; Elbrecht et al., 2017). Other biases are related to (c) the amount of DNA of the target organisms in samples that, albeit observed in tissue‐based metabarcoding approaches, may have a greater impact in preservative solutions driven by DNA release behaviours of organisms to the medium. When concentration of its DNA is very low, a taxon may be missed during preservative subsampling for DNA extraction or during the PCR amplification step, and so its detection probability would be low. Therefore, detection probabilities from preservative should be higher for taxa that are abundant and/or have high biomass than for taxa that are rare and/or have low biomass, since the DNA in solution will be more concentrated for the former than the latter (Carew et al., 2018; Erdozain et al., 2019; Hajibabaei et al., 2012). The amount of DNA in solution for a given taxon may also be conditioned by the body characteristics of individuals (Carew et al., 2018). Organisms that are soft bodied (e.g., Oligochaeta) may readily release cells or free DNA to the surrounding solution, and thus have higher probability of detection than organisms possessing a heavy chitinous exoskeleton (e.g., adults of Coleoptera), or with soft tissues protected by a shell (e.g., Mollusca) or a case (e.g., some Trichoptera; Carew et al., 2018; Zizka et al., 2019). The detection probabilities may also be lower for small‐bodied organisms since they have a smaller body surface in contact with solution compared to larger‐bodied organisms, though this has not been detected in previous studies (Carew et al., 2018; Erdozain et al., 2019; Zizka et al., 2019). Although these problems have been previously identified, their impacts on the results of metabarcoding from preservative solutions still need to be quantified in detail.

**TABLE 1:** Review of potential errors and biases in taxonomic recovery from metabarcoding of solutions preserving freshwater macroinvertebrate bulk samples, identified in this and previous studies

	Method‐specific ^a^	Description	References
Technical biases			
Marker adequacy			
(i) Reference databases	No	Certain taxa are still missed because they are not well represented in reference databases	Erdozain et al. (2019); this study
(ii) Primer specificity	No	“Universal” COI markers tested so far often miss non‐Insecta taxa (Mollusca, Annelida and Platyhelminthes), even when more than one primer set is used	Carew et al. (2018), Hajibabaei et al. (2012) and Zizka et al. (2019); this study
(iii) Bioinformatics	No	Certain taxa are discarded during attribute filtering by size in COI (e.g., Platyhelminthes, Mollusca and Diptera)	this study
Time of bulk fixation	Yes	Shorter periods (e.g., 1–5 days) decrease DNA yields and reduce taxonomic recovery	Martins et al. (2019)
Preprocessing steps	Yes	Taxonomic recovery increases when applying whole sample freezing (liquid nitrogen) but decreases when using the first ethanol phase in which specimens were preserved after sampling	Zizka et al. (2019)
DNA extraction	Yes	Mechanical lysis is less efficient in recovering taxa than enzymatic lysis, and DNA is likely to be captured more efficiently by magnetic beads than by column‐based methods	Martins et al. (2019)
Biological biases			
Abundance/biomass	no	Rare taxa or taxa with low biomass (e.g., Ephemeroptera, Trichoptera) are often missed	Erdozain et al. (2019) and Hajibabaei et al. (2012); this study
Body size	no	Small taxa (e.g., Hydrophilidae, Trombidiformes) are less likely to be detected	Carew et al. (2018) and Zizka et al. (2019)
Body structure			
(i) Sclerotization	Yes	More sclerotized taxa (e.g., Hemiptera, Coleoptera, Amphipoda) are often missed likely because they release less DNA to the medium	Carew et al. (2018) and Zizka et al. (2019); this study
(ii) Protective cases	Yes	Cased and shelled forms (e.g., certain Trichoptera families, Mollusca) are often missed compared to noncased sister groups	Carew et al. (2018) and Zizka et al. (2019); this study

^a^ Indicates which biases are mainly associated with metabarcoding of preservative samples.

In this study, we assessed biases in the taxonomic recovery of freshwater macroinvertebrates from the metabarcoding of ethanol used to preserve unsorted bulk samples, and we evaluated how they may affect the practical application of this approach in monitoring programmes. First, we assessed the adequacy of different markers to recover targeted taxa using both in silico analysis and a mock community sample (positive control), and then, we quantified metabarcoding biases using a realistic setting, involving 80 samples collected across Central Portugal in the scope of a national WFD monitoring programme. In the latter analysis, we compared detections of each taxon using metabarcoding and morphological identification, and applied a modelling approach to (a) estimate variation in probabilities of detection of each taxon in relation to per‐sample sequencing depth, and (b) relate probabilities of detection to absolute and relative abundances, sample richness, and to the body characteristics of each taxon, while controlling for variations in sequencing depth. To further test whether biases were conditional on metabarcoding markers, analyses were carried out in three genomic regions commonly used in the study of benthic macroinvertebrates: the mitochondrial cytochrome oxidase I (namely 5′‐COI region), the mitochondrial 16S rRNA and the nuclear 18S rRNA genes. Finally, we produced a multimarker data set by combining information from the three markers, which is expected to overcome some of the limitations of each individual marker (Cowart et al., 2015; Stefanni et al., 2018; Wangensteen, Palacín, Guardiola, & Turon, 2018). Our results show that metabarcoding of preservative ethanol produces strong bias against rare and hard‐bodied taxa, which needs to be overcome if this approach is to be adopted in monitoring programmes.

## 2 METHODS

### 2.1 Macroinvertebrate sampling

The study was carried out in central Portugal, over an area of about 11,215 km^2^ encompassing the watersheds of the Vouga, Mondego and Lis rivers (details in Mendes, Calapez, Elias, Almeida, & Feio, 2014; Mortágua et al., 2019). Bulk samples were obtained in the spring of 2017 at 80 river sites, following the Portuguese official protocols for collection of benthic macroinvertebrates in lotic systems (INAG, 2008). Briefly, a 50‐m sector of stream was selected at each sampling site, and six macroinvertebrate subsamples were collected by kick‐sampling using a kick‐net with 0.25‐m^2^ opening and 500‐µm mesh size, and covering the most representative habitats proportionally to their area in the sector. Each subsample involved kick/sweep sampling of 1‐m stream length in the upstream direction. All subsamples within a site were pooled into a single bulk sample, preserved in nondenatured 96% ethanol (UN1170) with an approximate ethanol:bulk ratio of 3:1 and stored at room temperature.

### 2.2 Laboratory procedures

#### 2.2.1 Morphotaxonomy

Macroinvertebrate samples were sorted and identified taxonomically at family level by an experienced researcher habilitated to process samples collected under the WFD. The number of individuals of each taxon in each sample was recorded. Identification was carried out at the family level because this is the resolution used in Portugal to estimate WFD‐based Ecological Quality Indices for freshwater benthic macroinvertebrates (INAG, 2008).

#### 2.2.2 Metabarcoding

For metabarcoding, we used the 80 samples collected in the field and a positive control with a mix of DNA extracted from specimens representing 55 macroinvertebrate families commonly recorded in our study area (Table S1). After careful manual shaking, 10 ml of preservative ethanol was taken from each macroinvertebrate bulk sample around 20 days after field sampling and transferred to an individual 15‐ml falcon tube where they were stored at −20ºC until DNA extraction. Prior to DNA extraction, a 2 ml subsample was taken from each falcon tube and ethanol was completely evaporated using an Eppendorf vacuum concentrator. Genomic DNA was then extracted with the column‐based E.Z.N.A.^®^ Tissue DNA Kit protocol (Omega BioTek, Inc.), using the InhibitEX^®^ Buffer (QIAGEN) instead of the manufacturer's lysis buffer, as described in Martins et al. (2019). At the final elution step, DNA was recovered from the spin column using 70 μl Elution Buffer incubated for 5 min at room temperature before the final centrifuge, diluted 1:2 (DNA extract:total volume) with ultrapure water and transferred to a microplate. Although E.Z.N.A.^®^ was not the best extraction method identified by Martins et al. (2019), we used it because it performed close to the best in ethanol samples taken >10 days after field sampling, and it was easier to implement for processing a large number of samples. Extractions were performed in batches of 24, including one extraction negative control, on a vacuum manifold (QIAGEN) to minimize cross‐contamination. Extraction replication was not performed due to the low variability in taxa composition among replicates found by Martins et al. (2019).

For PCR amplification, we used one marker per each of three genomic regions, which together were expected to detect most macroinvertebrate taxa: COI, 16S and 18S (Table 2). A COI marker (COI‐M19BR2) was used because this gene is commonly recommended for metazoans due to its high taxonomic resolution, lower taxonomic inflation and comprehensive reference databases (Andújar, Arribas, Yu, Vogler, & Emerson, 2018; Clarke, Beard, Swadling, & Deagle, 2017; Flynn, Brown, Chain, Macisaac, & Cristescu, 2015), although it contains poor conserved regions for suitable primer design, which may contribute to strong amplification biases (Deagle, Jarman, Coissac, Pompanon, & Taberlet, 2014; Elbrecht & Leese, 2017b). COI‐M19BR2 was found previously to consistently amplify Ephemeroptera, Plecoptera, Trichoptera and Odonata (EPTO) taxa (Martins et al., 2019), which are widely considered the best macroinvertebrate indicators of freshwater biological quality (Bonada, Rieradevall, Prat, & Resh, 2006). Although we also tested the widely used COI‐BF1BR1 (Elbrecht & Leese, 2017b) to enhance comparability with other studies, it was later discarded due to amplification problems. A 16S marker (16S‐Inse01) was used because this gene comprises more conserved regions than COI, reducing the chance of primer‐template mismatches and is thus expected to exhibit lower taxonomic bias (Clarke, Soubrier, Weyrich, & Cooper, 2014; Deagle et al., 2014), though it provides lower taxonomic resolution and in many geographic regions it is less represented in reference databases (Clarke et al., 2014; Elbrecht et al., 2016). 16S‐Inse01 was expected to provide high detection ability for a wide range of insect taxa (Taberlet, Bonin, Zinger, & Coissac, 2018), which usually dominate freshwater macroinvertebrate samples. Finally, we used an 18S marker (18S‐Euka02) due to the ability of this gene to screen a wide range of metazoan groups (Chain, Brown, Macisaac, & Cristescu, 2016; Zhan, Bailey, Heath, & Macisaac, 2014), although it may have relatively low taxonomic resolution for certain groups. 18S‐Euka02 marker was expected to allow the detection of non‐Arthropoda phyla (e.g., Platyhelminthes, Annelida, Mollusca; Taberlet et al., 2018), which are also common in freshwater macroinvertebrate samples.

**TABLE 2:** Description of metabarcoding markers used

Gene‐marker	Length range (bp)^b^	Primer name	Primer sequence (5′−3′)	Reference
COI‐M19BR2	305–322	Martins‐2019‐COI_Fw	GGNTGAACHGTHTAYCCHCC	Martins et al. (2019)
		BR2	TCDGGRTGNCCRAARAAYCA	Elbrecht and Leese (2017b)
COI‐BF1BR1^a^	—	BF1	ACWGGWTGRACWGTNTAYCC	Elbrecht and Leese (2017b)
		BR1	ARYATDGTRATDGCHCCDGC	Elbrecht and Leese (2017b)
16S‐Inse01	70–197	Inse01_F	RGACGAGAAGACCCTATARA	Taberlet et al. (2018)
		Inse01_R	ACGCTGTTATCCCTAARGTA	Taberlet et al. (2018)
18S‐Euka02	68–202	Euka02_F	TTTGTCTGSTTAATTSCG	Guardiola et al. (2015)
		Euka02_R	CACAGACCTGTTATTGC	Guardiola et al. (2015)

^a^ The COI‐BF1BR1 marker was initially used, but it was later discarded due to low amplification success.

^b^ Amplicon length range given by in silico amplification (see Section 2) for target families and incorporated into the bioinformatic pipeline.

PCR amplifications of 16S‐Inse01 and 18S‐Euka02 were made at the Laboratoire d’Ecologie Alpine (LECA, France). Each 20‐μl PCR contained 10 μl of AmpliTaq Gold Master Mix (Applied Biosystems™), 0.2 μM of each indexed primer, 5.84 μl ultrapure water, 0.04 μg of bovine serum albumin (Roche Diagnostic and 2 μl of DNA diluted 1:20 in total. After an initial denaturation cycle at 95°C for 10 min, 45 cycles (16S‐Inse01) or 50 cycles (18S‐Euka02) of 30 s at 95°C, 30‐s annealing at 52°C (16S‐Inse01) or 45°C (18S‐Euka02) and 60‐s extension at 72°C were performed, followed by a final elongation at 72°C for 7 min. Number of cycles and the dilution ratio were determined through qPCR beforehand to improve amplification success in these two markers. All PCR amplifications were performed in quadruplicate, including the positive control (i.e., mock sample) and three types of negative controls (tag, extraction and PCR; plate scheme: Figure S1). PCR products were pooled and sent to Fasteris SA for library preparation using the MetaFast protocol (www.fasteris.com/metafast; Taberlet et al., 2018). PCR amplification of COI‐M19BR2 was performed at CIBIO Laboratories following the two‐step protocol described in Martins et al. (2019) with few modifications. For the first‐round PCR, each 10‐μl PCR contained 5 μl of Hotstart Master Mix (Multiplex PCR Kit, QIAGEN), 0.4 μM of each primer, 2.2 μl ultrapure water and 2 μl of DNA diluted 1:2 in total. After an initial denaturation cycle at 95°C for 15 min, 40 cycles of 30 s at 95°C, 60‐s annealing at 50°C and 30‐s extension at 72°C were performed, followed by a final elongation at 60°C for 10 min. In the second‐round PCR, unique dual indexes (Gansauge & Meyer, 2013) were selected for each replicate and each 10‐μl indexing PCR contained 5 μl 2× KAPA HiFi HotStart ReadyMix (Kapa Biosystems), 5 μM of mixed indexing primer, 2 μl ultrapure water and 2 μl of 10× diluted first‐round PCR product. Indexing thermal cycling conditions were 95°C, for 3 min; followed by 10 cycles of 95°C for 30 s, 55°C for 30 s, 72°C for 30 s, with an extension of 72°C for 5 min. PCR products were validated through electrophoresis and pooled without normalization, similarly to the other two markers. Paired‐end (PE) sequencing was carried out in two separate Illumina (Illumina) runs: COI‐M19BR2 library was sequenced in a single run on an Illumina MiSeq system (2 × 250 bp), while the 18S‐Euka02 and 16S‐Inse01 libraries were sequenced in a single run on a HiSeq 2500 sequencing platform (2 × 125 bp) in Fasteris SA.

### 2.3 Bioinformatic Analysis

#### 2.3.1 Evaluation of marker adequacy

We conducted in silico analyses to assess the adequacy of each marker to amplify each of 98 macroinvertebrate families expected to occur in our study area. These analyses were undertaken using a script (Figure S2a) that combines custom‐built databases extracted from GenBank, in silico amplification using ecoPCR and informative outputs, enabling the user to test metabarcoding markers, evaluate their coverage in the database and measure their adequacy to target taxa. Databases for each marker consisted of sequences that met the following criteria: (a) they corresponded to the target amplicon region; (b) they contained the anticipated primer binding sites; and (c) they extended beyond primer binding sites. Criteria (a) and (b) were implemented to reduce bias in results, since running an in silico PCR without first confirming that all sequences correspond to the target amplicon region can produce false negatives (as noted by Egeter et al., 2019). The purpose of requirement (c) was to avoid having sequences that may not have had primers removed prior to being made public, which is a known issue with public barcoding sequences (Elbrecht & Leese, 2017a). The foundation for each database was created by downloading the entire GenBank database (October 2019) in GenBank format and extracting sequences matching the target genes (COI, 16S or 18S) according to the provided annotations. Taxonomy was added to sequences using taxonkit (Shen & Xiong, 2019), and those that did not have at least family‐level information, or did not belong to Metazoa, were removed. Sequences that did not correspond to the target amplicon region were removed as follows: (a) primer binding sites were located using ecopcr (Ficetola et al., 2010) with the maximum number of base mismatches set to 3 and the output set to include 5 bp either side of the amplicon; (b) amplicons with Ns or ambiguities were removed, to avoid complications in subsequent steps; (c) sequences for which primer binding sites were not found were mapped against sequences in which primer binding sites were found using the megablast algorithm (Zhang, Schwartz, Wagner, & Miller, 2000); (d) if the best alignment for a query sequence did not fully overlap the target amplicon region of the subject sequence, it was removed. Once the final databases had been created, ecoPCR was run again with the maximum number of base mismatches set to 5 and constraining maximum insert length per marker, accordingly to the Illumina sequencing chemistry used in the preservative samples (Figure S2). The ecoPCR results were compared against the final databases to identify which families were in silico‐amplified and which were not. To assess the informativeness of the target barcodes, the proportion of unique barcodes that could disambiguate at family level was calculated for each family by grouping any identical barcodes by their lowest common ancestor and checking the rank of that ancestor. Finally, the minimum and maximum amplicon lengths were retrieved per family to support read attribute processing.

#### 2.3.2 Sequence data processing

Sequence reads were processed using the obitools program suite (Boyer et al., 2016) (workflow in Figure S2b). After removing singletons, unique sequences of each marker were filtered by length. The amplicon length ranges, assessed using the in silico analyses, were used to maximize the detection of targeted families from read data (Table 2; Figure S3). Clustering was performed using sumaclust algorithm (Mercier, Boyer, Bonin, & Coissac, 2013) at 99% similarity for all markers. To remove potential false positives due to cross‐contamination, additional sequence filtering was performed using an integrated workflow adapted from Corse et al. (2017; LFN filtering). The filtering was based on the sequence variants found in each set of experimental controls, and LFN thresholds were calculated for each amplicon library. Specifically, based on the sumaclust output table, we filtered the sequence variants found in (a) the tag negative controls (LFNtag), discarding variants with counts lower than 0.003% per‐sample replicate; (b) extraction and PCR‐negative controls (LFNneg), subtracting the maximum absolute abundance of each variants found across negatives from each sample replicate count; and (c) positive controls (LFNpos), discarding variants from samples if (a) total abundance in positives was higher than 10% of total abundance across sample replicates and (b) maximum abundance in positives was higher than 10% of maximum abundance across sample replicates. Thresholds in (c) were estimated by visually checking expected contamination in wells containing nearby positive controls. We only kept sequence variants that passed all three LFN filters (one‐out all‐out strategy). Head clusters (cluster_center = True) were then retrieved, and sequence counts of variants were summed to respective heads. Simultaneously, to assess amplification bias among markers across the 55 known families pooled, we retrieved the sequence count of head clusters observed in the positive controls. Since PCR products were pooled without normalization before sequencing and the sequence counts varied substantially within samples (a.k.a. sampling sites), the four technical replicates were combined prior to analysis.

#### 2.3.3 Taxonomic assignment

The taxonomic assignment procedure followed Martins et al. (2019), using public and in‐house reference databases: COI‐M19BR2 (BOLD, GenBank, CIBIO‐IBI and aquaDNA), 16S‐Inse01 (GenBank, CIBIO‐IBI and aquaDNA) and 18S‐Euka02 (GenBank and aquaDNA). CIBIO‐IBI is an in‐house database containing mainly species from the Iberian Peninsula (Ferreira et al., 2018), which will be published in due course (e.g., Ferreira et al., 2020). AquaDNA is an in‐house database including mainly French species Ficetola et al., 2020, though it covers many families occurring in Iberia. After manual cross‐validation, only clusters assigned to macroinvertebrate taxa at ≥92% identity in at least one database were kept and clusters associated with multiple hits were assigned back to the taxonomic rank of the common ancestor. All assignments were manually curated to account for species ranges, with species not recorded in the study area being (a) replaced by those known to occur, in the case of single‐species genera, (b) kept if assigned with a high percentage of identity to species of less‐studied groups (e.g., Chironomidae, Platyhelminthes, Oligochaeta) or (c) assigned to genus in case of diverse genera. A final taxon table was created by merging sequence counts by family, removing rare occurrences (<0.01%) within each sampling site by marker and converting counts into presence/absence values. The filtering of rare taxa was applied at the family level to avoid discarding families represented by several rare cluster sequences. Families belonging to Oligochaeta and Hydracarina were merged into these two groups, respectively, since this was the level of identification that is generally achieved through morphotaxonomy. A “multimarker” data set was created by combining information of the three markers in each sampling site, that is, a family was detected by multimarker if recovered by at least one marker. A single phyloseq object (r phyloseq package, McMurdie & Holmes, 2013) combining the three matrices (taxa, sample and taxonomy) of each individual marker, multimarker and morphotaxonomy was created to facilitate further analysis.

### 2.4 Statistical analyses

As in Martins et al. (2019), we used morphotaxonomy as the benchmark for molecular data instead of metabarcoding the bulk sample itself because (a) our bulk samples were collected under the national WFD monitoring programme and thus could not be destroyed; (b) we wanted to evaluate how abundance (i.e., number of individuals) in the bulk sample affected the probabilities of detection of each family, and this information could not be obtained by metabarcoding the bulk sample; and (c) we wanted to compare metabarcoding with the standard morphological approaches used in WFD monitoring. In all analyses, we considered for each marker and sampling site the families recorded by both molecular and morphotaxonomic methods (coded as 1), and families that were detected through morphotaxonomy but not by the molecular method (coded as 0). The analyses thus concentrated on “true” positives (1) versus “false” negatives (0), ignoring families that were only detected through molecular methods (i.e., “false” positives). This approach assumes that there were no errors in the identification of taxa through morphotaxonomy, which is a reasonable assumption because identifications were made at the family level, which are readily identifiable by experienced taxonomists. We did not address false positives because this would require different experimental procedures, controlling for various sources of error including contamination, regurgitation of digested material by individuals in the bulk sample and/or taxonomic misidentifications (Martins et al., 2019; Zizka et al., 2019).

We first assessed variation between methods in the diversity and taxonomic composition of macroinvertebrate communities retrieved through individual markers, the multimarker approach and morphotaxonomy. Local (i.e., alpha) diversity was estimated using the estimate_richness function (r phyloseq package), as the number of families observed per sampling site using each method. To help interpret differences in richness, we also estimated the percentage of matching families recorded at each sampling site between each pair of methods, by computing the quotient between the number of families shared by both methods in relation to the total family richness detected by those methods at the sampling site. The extent of change in community composition among sampling sites (i.e., beta diversity) was computed for each method using the pairwise Jaccard coefficient of dissimilarity in the vegan r package (Oksanen et al., 2019). We also evaluated whether beta diversity estimates were consistent across methods, using Procrustes analysis (vegan r package) to compare the Jaccard dissimilarity matrices for each pair of methods. Additionally, we estimated variation in the taxonomic composition retrieved by the different methods using differential heat trees, produced with the metacoder r package (Foster, Sharpton, & Grünwald, 2017). To build the heat trees, we considered that the representation of each family was proportional to the number of sequences in the case of molecular methods and the number of individuals for morphotaxonomy. Although the number of sequences does not necessarily equate to the relative abundance or biomass of each family (Elbrecht & Leese, 2015), this approach was taken to obtain a visualization of how broad taxonomic branches were missed or retrieved by the different methods.

To model detection biases, we first used binomial logit generalized linear models (i.e., logistic regression) to relate the probability of detection of each family using a given marker in relation to read abundance per sample (i.e., read counts after demultiplexing step). This data set was used instead of the clean data set described above (i.e., filtering sequences assigned to families detected by morphotaxonomy), because per‐sample sequencing depth is a parameter that researchers can control when setting up sequencing assays, whereas final sequence abundance depends on data processing. However, results were largely similar irrespective of the data set used (not shown). In the case of the multimarker, we simulated read abundance by summing reads across markers. Because the detection is necessarily zero when sequencing depth is zero, we forced in all cases the logistic model intercept at nearly zero by including a suitable offset term. This preliminary analysis was needed because sequencing depth varied widely across sampling sites and markers, and so the proportion of true positives computed directly from the data would not be comparable across markers and families. To overcome this problem, we thus used the logistic models to normalize the probability of detection to a sequencing read count of 50,000 for each family and marker.

We further analysed factors affecting detection probabilities across families and sampling sites using binomial logit generalized linear mixed models (GLMM) for each marker data set and multimarker. In the fixed component of the model, we included for each family (a) the total number of individuals and (b) the proportion of individuals at each sampling site (i.e., number of individuals of the family divided by the total number of individuals of all families observed in the sample), and (c) the “body armouring” trait as described by Poff et al. (2006). Families were categorized into one of the three described armouring categories (Table S3): soft‐bodied (e.g., Annelida, Ephemeroptera), heavily sclerotized (e.g., Coleoptera) and cased forms (e.g., Mollusca and some Trichoptera families). For each sampling site, we also included (d) the total number of recorded families and (e) the sequencing depth (log10‐transformed). Sampling site and family were incorporated in the models as random effects. Count and proportional data were scaled prior to analyses, and sampling sites without families detected were removed for each marker. Taxonomic groups not detected in silico or not amplified by the 16S‐Inse01 marker (Platyhelminthes and cased families) were excluded from the analysis to allow model convergence.

## 3 RESULTS

### 3.1 Morphotaxonomy

Overall, the 80 macroinvertebrate bulk samples yielded 74,335 individuals of 94 families belonging to four phyla, with an average (±*SD*) of 929.2 ± 998.3 (55–7,908) individuals and 23.8 ± 9.5 (4–41) families per sample (Tables S2 and S3). The most widespread families (occurrence in ≥50% of samples) were Chironomidae (100.0%), Baetidae (93.8%), Oligochaeta (91.3%), Simuliidae (90.0%), Elmidae (80.0%), Hydropsychidae (77.5%), Ephemerellidae (75.0%), Caenidae (68.8%), Ceratopogonidae (65.0%), Leptophlebiidae (57.5%), Rhyacophilidae (53.8%), Hydrobiidae (52.5%) and Ancylidae (51.3%), while 31 families occurred in <10% of samples. The most abundant families (>5% of individuals) were Hydrobiidae (17.7%), Chironomidae (17.6%), Simuliidae (12.2%), Ephemerellidae (10.7%), Baetidae (9.3%) and Elmidae (5.3%). Most families (*N* = 80) had less than 1% of individuals, of which 43 had each <0.1% of individuals.

### 3.2 Marker adequacy

The in silico analysis revealed higher marker adequacy for 16S‐Inse01 (87.8% of families amplified; *n* = 98) than COI‐M19BR2 (82.7%) or 18S‐Euka02 (71.4%; Table 3; Table S4). The majority of failures in COI‐M19BR2 was due to the lack of primer binding sites in the existing barcodes, whereas in 16S‐Inse01, it was due to complete lack of barcodes (i.e., target insert). Regarding 18S‐Euka02, there were families failing due to lack of primer binding sites in barcodes (13.3%), but also due to lack of amplification (11.2%) and resolution (4.1%). The main taxonomic groups with failures (<80% families amplified) for COI‐M19BR2 were from the orders Coleoptera, Diptera, Heteroptera, Ephemeroptera and Trichoptera, for 16S‐Inse01 it was Trichoptera, and for 18S‐Euka02 were noninsect arthropods, Diptera, Trichoptera and Odonata (Table 3).

**TABLE 3:** Summary results of in silico and in vitro analysis for three metabarcoding markers (COI‐M19BR2, 16S‐Inse01 and 18S‐Euka02) to detect the main freshwater macroinvertebrate families recorded in Central Portugal

	In silico					In vitro	Failed only in vitro (%)	Failed only in silico (%)
	Marker adequacy (%)	Detection failures				Marker adequacy (%)		
		Barc	Bind	Amp	Res			
COI‐M19BR2								
Overall	82.7	1.0%	16.3%	—	—	89.1	9.1	10.9
Annelida	100.0	—	—	—	—			
Mollusca	87.5	—	12.5%	—	—			
Platyhelminthes	100.0	—	—	—	—			
Noninsect arthropods	83.3	—	16.7%	—	—			
Coleoptera	75.0	—	25.0%	—	—	77.8	11.1	11.1
Diptera	77.8	—	22.2%	—	—	80.0	20.0	10.0
Hemiptera	71.4	14.3%	14.3%	—	—	100.0	0.0	20.0
Megaloptera	100.0	—	—	—	—	100.0	0.0	0.0
Ephemeroptera	75.0	—	25.0%	—	—	100.0	0.0	0.0
Plecoptera	100.0	—	—	—	—	100.0	0.0	0.0
Trichoptera	78.9	—	21.1%	—	—	93.8	6.3	18.8
Odonata	100.0	—	—	—	—	85.7	14.3	0.0
16S‐Inse01								
Overall	87.8	12.2%	—	—	—	44.4	38.9	0.0
Annelida	100.0	—	—	—	—			
Mollusca	100.0	—	—	—	—			
Platyhelminthes	100.0	—	—	—	—			
Noninsect arthropods	83.3	16.7%	—	—	—			
Coleoptera	100.0	—	—	—	—	11.1	88.9	0.0
Diptera	100.0	—	—	—	—	40.0	60.0	0.0
Hemiptera	100.0	—	—	—	—	60.0	40.0	0.0
Megaloptera	100.0	—	—	—	—	100.0	0.0	0.0
Ephemeroptera	100.0	—	—	—	—	66.7	33.3	0.0
Plecoptera	100.0	—	—	—	—	100.0	0.0	0.0
Trichoptera	42.1	57.9%	—	—	—	20.0	20.0	0.0
Odonata	100.0	—	—	—	—	85.7	14.3	0.0
18S‐Euka02								
Overall	71.4	13.3%	—	11.2%	4.1%	47.2	32.1	5.7
Annelida	100.0	—	—	—	—			
Mollusca	87.5	—	—	—	12.5%			
Platyhelminthes	100.0	—	—	—	—			
Noninsect arthropods	50.0	—	—	33.3%	16.7%			
Coleoptera	91.7	—	—	8.3%	—	44.4	44.4	0.0
Diptera	44.4	16.7%	—	38.9%	—	0.0	50.0	0.0
Hemiptera	100.0	—	—	—	—	100.0	0.0	0.0
Megaloptera	100.0	—	—	—	—	0.0	100.0	0.0
Ephemeroptera	100.0	—	—	—	—	100.0	0.0	0.0
Plecoptera	100.0	—	—	—	—	75.0	25.0	0.0
Trichoptera	42.1	52.6%	—	5.3%	—	43.8	25.0	18.8
Odonata	75.0	—	—	—	25.0%	42.9	42.9	0.0

Note

For each marker and taxonomic group, we provide the percentage of families adequately detected (Marker adequacy). For the in silico analysis (based on GenBank database), we provide the percentage of families failing due to the lack of barcodes (barc) or of primer binding sites in barcodes required for testing (bind), no amplification (amp) or no resolution (res). We also present the percentages of families detected in silico but not in vitro (Failed only in vitro), and the percentage of families detected in vitro but not in silico (Failed only in silico). The mock sample used in vitro is described in Table S1.

In contrast to in silico results, analysis of the positive control underlined a much better marker adequacy for COI‐M19BR2 (89.1% of families amplified; *n* = 55) than for either 16S‐Inse01(43.6%) or 18S‐Euka02 (45.5%; Table 3). COI‐M19BR2 successfully detected most families (>80%) except Coleoptera, while 16S‐Inse01 and 18S‐Euka02 had low detection rates (<80%) except for Megaloptera and Plecoptera, and Heteroptera and Ephemeroptera, respectively. Considering taxa that were tested both in silico and in vitro, the differences for the latter two markers were due to a large proportion of families that amplified in silico but not in vitro (16S‐Inse01 = 38.2%; 18S‐Euka02 = 30.9%), while there were only a few families failing in silico but amplifying in vitro (16S‐Inse01 = 0.0%; 18S‐Euka02 = 5.5%) due to the lack of barcodes in the database. For COI‐M19BR2, the two types of errors were similar (9.1% vs. 10.9%).

### 3.3 Sequencing data

Sequencing of libraries generated 25,232,044 PE reads for COI‐M19BR2, 50,185,936 PE reads for 16S‐Inse01 and 39,544,628 PE reads for 18S‐Euka02 (Table S5). Sequencing depth varied greatly across sampling sites within markers: COI‐M19BR2—10,620–662,342 reads; 16S‐Inse01—50,331–605,502 reads; and 18S‐Euka02—6,374–529,412 reads. After data processing, about half of the original read count was kept for 16S‐Inse01 (representing 26,262 clusters) and COI‐M19BR2 (10,146 clusters), and about 60% for 18S‐Euka02 (33,318 clusters; Table S5). More than 80%–90% of clusters generated by the three markers were identified with a percentage of identity ≥92%, mostly assigned at genus and family ranks (Table S5 and Figure S4). Rarefaction curves suggest that sample depth was enough for recovering all families in most preservative samples (Figure S5). The final data set, including only targeted macroinvertebrate families detected by morphotaxonomy, showed similar magnitudes of sequence abundance among markers (Table S5).

### 3.4 Variation in community diversity and composition across methods

**FIGURE 1:**
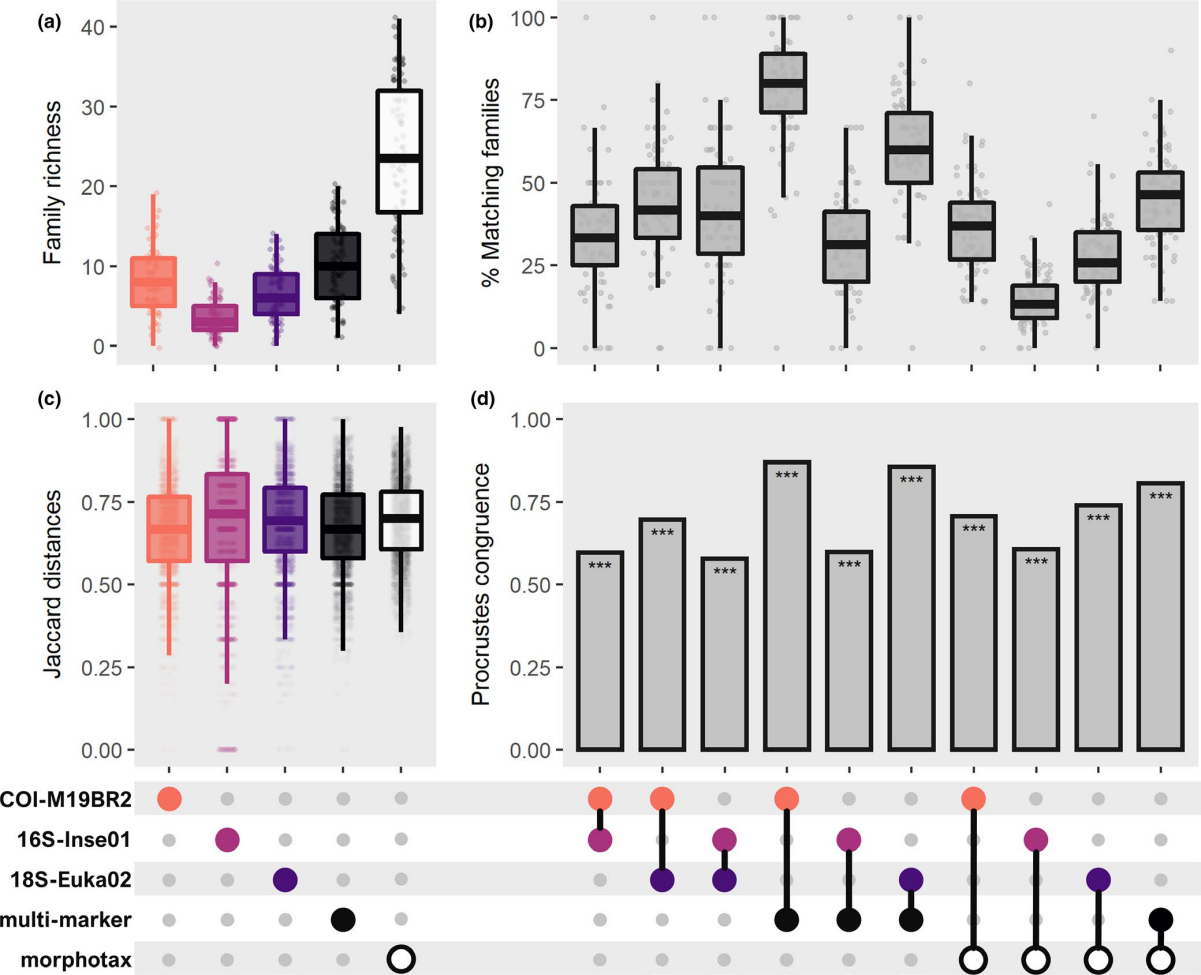
Comparisons of diversity metrics and taxonomic composition of freshwater macroinvertebrate samples, estimated using either morphotaxonomy, one of three molecular markers (COI‐M19BR2, 16S‐Inse01 or 18S‐Euka02), or a multimarker approach: (a) observed richness (alpha diversity), (b) taxonomic overlap, (c) Jaccard dissimilarity distributions between all pairs of sampling sites (beta diversity) and (d) taxonomic congruence in community composition (using Procrustes analysis). Only families detected by morphotaxonomy (sample‐wise) were considered. Boxplots represent median, 1st and 3rd quartiles, and extremes (1.5× interquartile range). Plots on panels b and d denote pairwise comparisons between methods

The average number of macroinvertebrate families per sampling site (alpha diversity) detected using each individual marker or the multimarker was always much lower than those detected using morphotaxonomy (Figure 1a). There was also a low match in family composition across molecular methods (Figure 1b), albeit much higher between multimarker and COI‐M19BR2 (79.3% ± 16.9). The match with morphotaxonomy was only low for 16S‐Inse01 (14.1% ± 7.6) and 18S‐Euka (27.6% ± 11.0). The distribution of Jaccard dissimilarities among sampling sites (pairwise beta diversity) was comparable across methods (Figure 1c), with Procrustes analysis revealing that the matrices of Jaccard dissimilarity among sampling sites were fairly consistent across methods (*r* > .70; Figure 1d), except for 16S‐Inse01. Therefore, sampling sites with high dissimilarity estimated through morphotaxonomy also tended to be estimated as highly dissimilar with metabarcoding, especially for multimarker (*r* > .80).

The differential heat trees revealed major differences in taxonomic composition of macroinvertebrate communities retrieved through the different methods (Figure S6). As expected, the proportions of reads assigned to Insecta (except for the Ephemerellidae family) were higher for COI‐M19BR2 and 16S‐Inse01 comparatively to 18S‐Euka02, whereas the latter recovered phyla hardly detected by the former such as Mollusca. There were also differences between the two mitochondrial markers, with 16S‐Inse01 recovering fewer Diptera, Trichoptera and Oligochaeta than COI‐M19BR2. There was low congruence on the proportion of abundance between markers (sequence counts) and morphotaxonomy (individuals), with higher ratios towards Mollusca and Insecta families in morphotaxonomy than in molecular methods.

### 3.5 Factors affecting variation in detection probability

**FIGURE 2:**
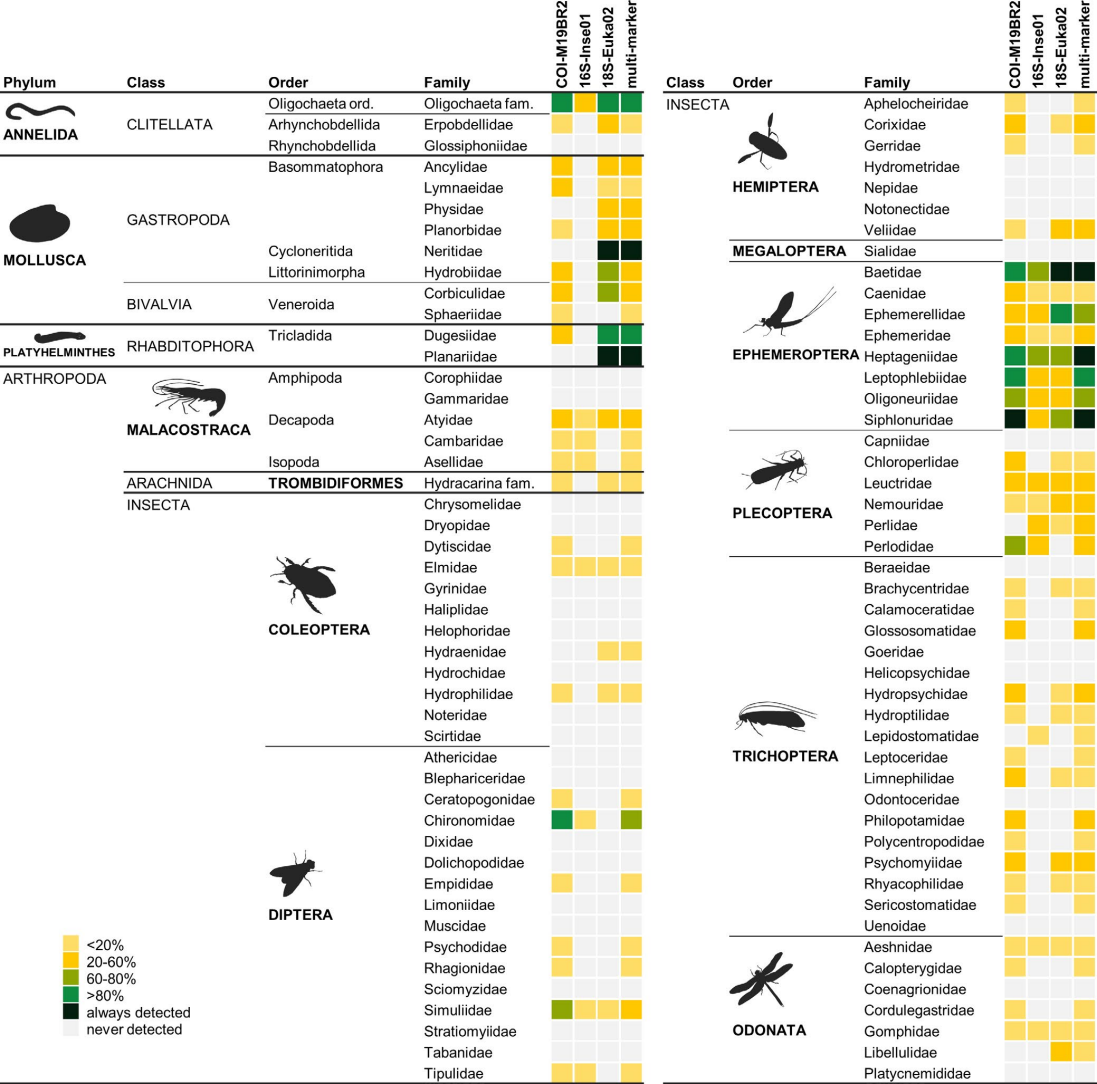
Detection probabilities of macroinvertebrate taxa recovered from the metabarcoding of ethanol preserving unsorted bulk samples (*N* = 80), using either one of three markers (COI‐M19BR2, 16S‐Inse01 or 18S‐Euka02) or a multimarker approach. Detection probabilities were normalized at a sequencing read count of 50,000, based on predictions from logistic regression models relating the presence–absence of each family with per‐sample sequencing depth (see Figure S8). Only families detected by morphotaxonomy (sample‐wise) were considered

As expected, sequencing depth per sample greatly affected the probabilities of detection of each macroinvertebrate family across sampling sites (Figure S7). After normalizing sequencing read count to 50,000, there were still major variations in detection probabilities across markers and taxonomic groups (Figure 2; Figure S8). Differences between markers are illustrated, for instance, by the detection probabilities of Trichoptera families, which were low (<20%) to moderate (20%–60%) for COI‐M19BR2, while they were not detected at all or were detected at low probabilities in 16S‐Inse01 and 18S‐Euka02. In contrast, the probabilities of detection were high (>80%) for Tricladida families using 18S‐Euka02, while they were not detected by the other two markers. Regarding taxonomic differences, it is noteworthy for instance that most families in the orders Coleoptera, Diptera, Hemiptera and Odonata were not detected or had very low detection probabilities for all markers, whereas Ephemeroptera and, to a lesser extent, Plecoptera families were generally detected with low to very high probabilities. There was also substantial variation in detection probabilities among different families of the same order, with for instance Chironomidae and Simuliidae detected with high probabilities by COI‐M19BR2, while other families were either not detected or detected at low probabilities irrespective of the marker. Likewise, in the order Ephemeroptera, the detection of Baetidae and Heptageniidae families was always high, irrespective of marker, while for instance Caenidae and Ephemeridae showed in general low detection probabilities.

When combining detections from the three markers in the multimarker data set, there were increases in detection probabilities for most taxonomic groups, though they were still low for Coleoptera and most Diptera, Odonata and Hemiptera families, among others. Only three families were always detected, namely Neritidae (Mollusca), Planariidae (Platyhelminthes) and Baetidae (Ephemeroptera) by 18S‐Euka02, Siphlonuridae (Ephemeroptera) by COI‐M19BR2, and Heptageniidae (Ephemeroptera) when the three markers were combined (multimarker).

**FIGURE 3:**
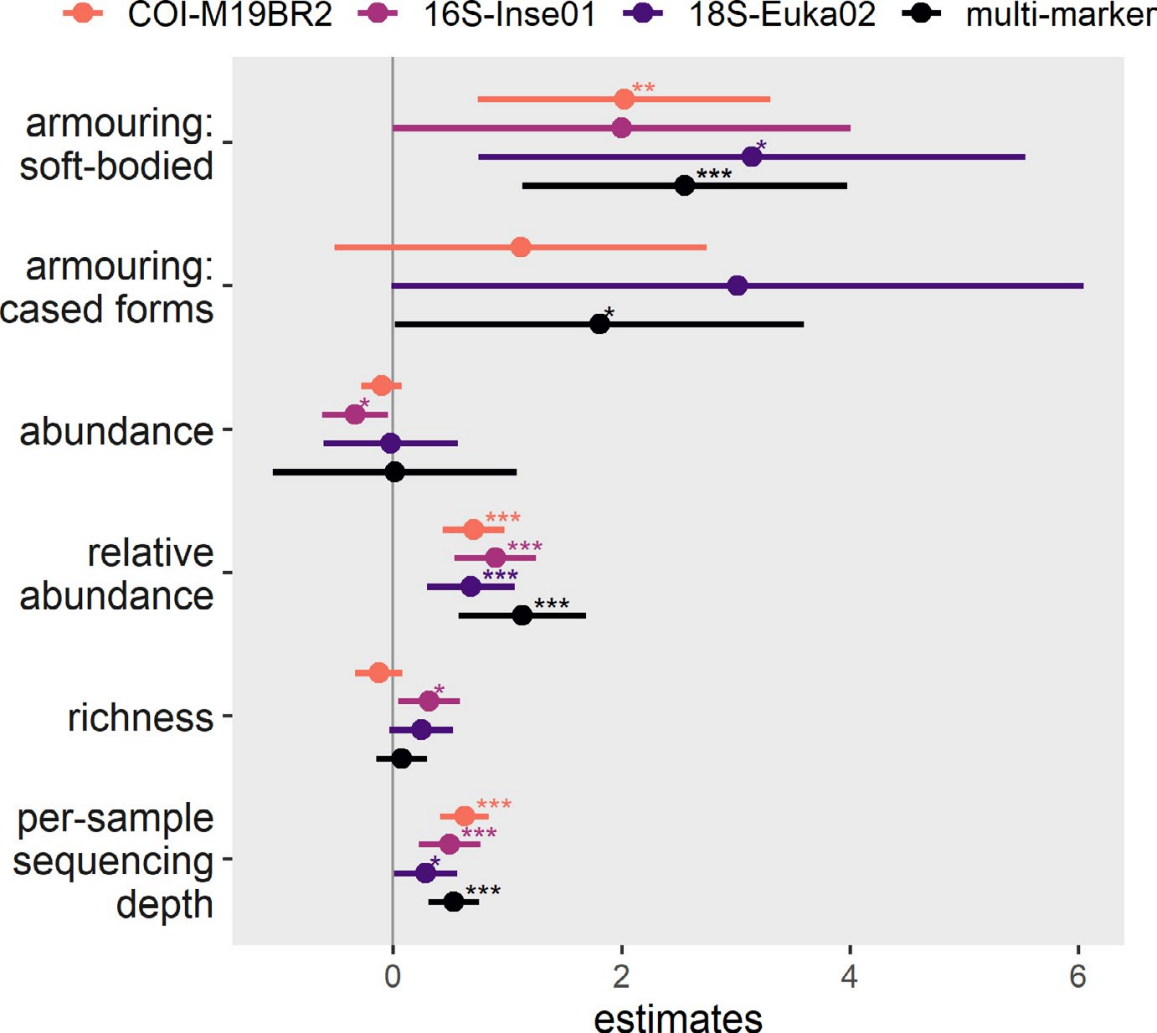
Summary results of generalized linear mixed models (GLMM) relating the probability of detection of freshwater macroinvertebrate taxa from metabarcoding of ethanol to variables describing its body armouring (Poff et al., 2006), total abundance (i.e., number of individuals in the bulk) and relative abundance (i.e., proportion of individuals) and to variables describing the taxa richness and the sequencing depth of the bulk sample. GLMMs were estimated separately for each marker (COI‐M19BR2, 16S‐Inse01 or 18S‐Euka02) and the multimarker approach. Values represented are estimated model coefficients (mean ± 95% confidence interval) and respective significance (***p < .001; **p < .01; *p < .05; Table S6). Only families detected by morphotaxonomy (sample‐wise) at least in five sampling sites were considered

After controlling for per‐sample sequencing depth, there were significant effects of body armouring and relative abundance on detection probabilities for all markers and the multimarker (Figure 3; Table S6). Probabilities of detection were consistently higher for soft‐bodied than heavily sclerotized families, while cased forms had intermediate values varying within markers. Detection probabilities of a family also increased markedly with its relative abundance in the sample, but there were no highly significant effects of its total abundance or family richness per sample.

## 4 DISCUSSION

This study highlighted important biases in taxonomic recovery when using metabarcoding from the ethanol used to preserve macroinvertebrate bulk samples. While the in silico analysis suggested that the markers used were adequate to detect our target macroinvertebrate taxa, the analysis of both the positive control (mock sample) and the 80 samples collected under a WFD monitoring programme showed that many taxa represented in the bulk were often missed in metabarcoding and that the probability of detection of each taxon often varied greatly among samples. Notwithstanding, the multimarker approach performed consistently better than any single marker, and the COI marker performed better than the two rRNA markers. In line with expectations (Table 1), our results suggest that detection problems were largely related to biases against the recovery of rare and sclerotized taxa, irrespective of the marker used. Specifically, our modelling approach showed that after controlling for variation in read coverage among samples, the probability of detection of any given taxon was positively related to its proportional abundance in the sample, and that the probability of detection was on average lower for heavily sclerotized taxa (e.g., Coleoptera) than for soft‐bodied taxa (e.g., Oligochaeta). Overall, these results suggest that further methodological development and optimization is needed before metabarcoding from ethanol can be used in biomonitoring programmes.

The in silico analysis provided important information to optimize the subsequent bioinformatic procedures and to evaluate marker adequacy. In particular, this analysis was used to calculate suitable amplicon length ranges for the target groups considering taxon‐specific amplicon variation (including primer slippage for degenerated primers; Elbrecht, Hebert, & Steinke, 2018), which provides a more robust approach than the usual practice of retrieving such information from similar published studies (e.g., Elbrecht et al., 2017; Taberlet et al., 2018; Wangensteen et al., 2018). We found this to be very important, especially for the COI‐M19BR2 marker, because certain families of Platyhelminthes, Mollusca and Diptera were missed when applying existing thresholds (e.g., 310–316 bp). Regarding adequacy, we found that the markers chosen were largely adequate for the macroinvertebrate communities targeted in our study, with >80% of taxa recovered for 16S‐Inse01 and COI‐M19BR2, though adequacy was lower (≈70% of taxa) for 18S‐Euka02. Detection failures in COI‐M19BR2 were due exclusively to either the lack of barcodes or barcodes with primer binding sites for some target taxa in the GenBank database, while detection failures in 16S‐Inse01 were only due to the lack of barcodes, which emphasizes the importance of comprehensive barcode reference databases for molecular biomonitoring (Weigand et al., 2019). In the case of 18S‐Euka02, besides failures due to barcode unavailability, there were also detection problems due to lack of amplification and, to a lesser extent, lack of resolution. This suggests that the 18S marker used in our study may be more prone to miss taxa than the other two, despite its expected ability to detect a wider taxonomic range of macroinvertebrate taxa (Taberlet et al., 2018).

In contrast to the in silico results, the analysis of the mock sample and of the samples collected under the WFD monitoring programme showed that many taxa detected in each sample through morphotaxonomy were often missed with metabarcoding, as reported elsewhere (Elbrecht et al., 2017; Erdozain et al., 2019). The detection ability was much higher for COI‐M19BR2 than for either 16S‐Inse01 and 18S‐Euka02, which emphasizes that despite codon degeneracy this marker may be very powerful (Clarke et al., 2017; Elbrecht et al., 2016). However, this result may not be a consequence of a superior performance by the COI marker in itself, but instead it may reflect differences between laboratories in the conditions used to amplify each individual marker to overcome PCR inhibition. While for COI‐M19BR2, the procedure involved a 1:2 dilution of the extract and using a polymerase (Qiagen Master Mix) that is less prone to inhibition and works well on ethanol samples at this dilution ratio, following previous optimization (Martins et al., 2019), for 16S‐Inse01 and 18S‐Euka02 the protocol involved a 1:20 dilution to enhance polymerase activity. These results clearly suggest that relatively small changes in protocols can have major consequences on metabarcoding results, though these dilutions were required to improve amplification success in each protocol. Despite these differences, all markers consistently underestimated the richness of taxa represented in the bulk, and there were incongruences across markers in the taxonomic composition of the community. As suggested in previous metabarcoding studies, these problems were minimized to some extent by combining information from the three markers (da Silva et al., 2019), though the multimarker approach still detected less taxa than morphotaxonomy. Interestingly, however, estimates of beta diversity were similar for morphotaxonomy, the three single markers and multimarker approach, which is consistent with results of a study by Clarke et al. (2017) comparing the performance of COI, 16S and 18S markers for zooplankton metabarcoding. This suggests that while metabarcoding may underestimate alpha diversity in relation to morphotaxonomy, it may still provide comparable results regarding the turnover of taxa across sites.

Irrespective of the marker used, the probability of detection of any given taxon in a sample was strongly affected by the read coverage of the sample, though there were still major differences in detection probability across taxa and samples after statistically controlling for the effect of read coverage. Our modelling results suggest that part of this variation was related to taxa abundance, with higher detection probability for more abundant taxa in the bulk. This effect was consistent across markers and for the multimarker approach. Comparable results have been found elsewhere (Table 1), though to the best of our knowledge this is the first time the effect of abundance is modelled in detail using large sample sizes. Usually, this result is interpreted assuming that abundance (or biomass) of an organism in the bulk is related to the concentration of its DNA in the preservative solution and that the probability of detection is directly related to its DNA concentration. This is because at low DNA concentrations a taxon may be missed during the subsampling of preservative ethanol or it may not be amplified during the PCR step due to “primer competition” with DNA of more abundant taxa. The later hypothesis is supported by our results, which showed a strongly significant effect of proportional abundance (i.e., the number of individuals of a taxa divided by the total number of individuals in the sample) on probability of detection, but not of total abundance (i.e., the total number of individuals). Other studies have also reported a lower detection for taxa represented in the bulk by a low proportion of individuals (Hajibabaei et al., 2012) or biomass (Erdozain et al., 2019). Similar problems have also been described in tissue‐based metabarcoding protocols (Elbrecht & Leese, 2015; Hajibabaei et al., 2012).

Besides abundance, our results also showed that body traits drive variations in probability of detection across macroinvertebrate taxa. Although we did not find the hypothesized effects of body size (Carew et al., 2018; Zizka et al., 2019), we did confirm that body sclerotization reduces the probability of detection, which had previously been observed in freshwater benthic macroinvertebrates (Carew et al., 2018; Zizka et al., 2019) and terrestrial invertebrates (Marquina, Esparza‐Salas, Roslin, & Ronquist, 2019). Specifically, we found that the detection probability was very low for heavily sclerotized arthropod taxa such as Coleoptera, possibly because body armouring reduced the release of DNA to the preservative solution, thereby causing problems of detection similar to those of taxa with low proportional abundance. Taxa with cased forms, which includes soft‐bodied organisms with a shell (Mollusca) or a case (caddisflies), showed intermediate recovery rates between soft‐bodied and sclerotized organisms. This suggests that the presence of a case also reduces the release of DNA, though not as much as in the case of heavily sclerotized organisms. It should also be noted that at least in some Trichoptera families the individuals leave the case after collection, which probably reduced the effect of this body trait on detection probabilities.

It is unlikely that these general results and key conclusions of our study were influenced to any significant extent by methodological artefacts or biases, though we recognize that the consequences of some options taken still need further research. One potential problem is the low volume of ethanol analysed, which may have contributed to missing rare taxa due simply to sampling effects. However, in a previous study using the same approach we found little variation in community composition across small ethanol subsamples, probably because they were consistently retrieving the commonest taxa (Martins et al., 2019). This suggests that adding small subsamples to obtain a larger volume analysed would have had only a modest effect on the number of taxa retrieved, though the effects of the volume of ethanol used on metabarcoding performance require further investigation. Moreover, our key results relating the probability of detection to body armouring and relative abundances are unlikely to be affected by the volume analysed, and they were consistent with the results of a study using much larger volumes (Zizka et al., 2019). Another potential problem is that we used a similar assignment threshold of 92% identity for the three markers, though this may be overly strict for markers in genomic regions with high substitution rate such as COI, and eventually too liberal for more conserved regions such as 18S rRNA gene. However, we believe the use of this common threshold is reasonable because: (a) there is a good evidence that the rates vary between taxa for the same marker (especially for ribosomal genes); (b) reducing the threshold below 92% might produce an unacceptable level of false positives, even for COI; (c) identifications for all markers were most often made at identity levels well above 92% (Figure S4), further supporting the view that small changes in threshold have limited effect on reducing false negatives, and (d) small reductions in the threshold (say, from 92% to 90% in COI) to limit false negatives would result in a small proportion of additional reads identified, most of which to taxa with high detection probabilities. It is possible, however, that decreasing the threshold would further improve the performance of the COI markers, though changes would likely be small.

The results of our study have consequences for the application of metabarcoding of preservative ethanol to the molecular biomonitoring of aquatic systems. First, the lower number of taxa detected per site (alpha diversity) with metabarcoding highlights that morphotaxonomy and metabarcoding results cannot be directly compared, though this can probably be solved through intercalibration exercises (Pawlowski et al., 2018). Second, the observation that morphotaxonomy and metabarcoding estimated similar turnover of taxa (beta diversity) across sampling sites is promising, suggesting that meaningful ecological gradients can be retrieved using either approach, which can provide a basis to detect anthropogenic stressors (e.g., Santana et al., 2017). Third, care should be taken to account for biases associated with the strong dependence of detection probability on proportional abundance, which implies that the probability of detection of a species depends not only on its abundance but also on the abundance of other species in the community (Erdozain et al., 2019; Hajibabaei et al., 2012), and thus that errors may be inconsistent across sampling sites and over time. Consequently, spatial and temporal variations detected through metabarcoding in the occurrence of rare indicator organisms may be unreliable, which can compromise their use in biomonitoring. Finally, there may be systematic problems in the detection of potentially good biodiversity indicators such as Coleoptera (Sánchez‐Fernández, Abellán, Mellado, Velasco, & Millán, 2006), given the low detection probability of heavily sclerotized arthropods.

In summary, our results suggest that, apart from barcode reference databases, the main biases associated with metabarcoding of preservative solutions are largely related to the differential availability of DNA from different organisms, which in turn is affected mainly by their body armouring and proportional abundance. These problems suggest that, depending on the application, it should be duly considered whether to use metabarcoding from the preservative solution or from the homogenate of the bulk itself, as the latter may be less prone to the biases identified here (Marquina et al., 2019; Zizka et al., 2019). However, while using tissue homogenates may eliminate the problem of detecting hard‐bodied organisms, it may still have biases associated with reduced detection of taxa with low proportional abundance (Elbrecht & Leese, 2015; Hajibabaei et al., 2012). When metabarcoding from the preservative solution is required to preserve the bulk or to reduce the problems associated with the sorting of specimens, then efforts should be made to enhance DNA recovery through optimized procedures considering, for example, the timing of preservative collection after field sampling (Martins et al., 2019), preprocessing measures such as whole sample freezing prior to filtering ethanol (Zizka et al., 2019), higher sampling volumes or bait capture enrichment (Gauthier et al., 2020). Also, the use of multiple markers is highly recommended, as it provides a much more comprehensive representation of the taxonomic composition of bulk samples, as found for instance in dietary studies (da Silva et al., 2019). The number and exact mix of markers to be used still needs further research, but our results suggest that a combination of COI‐M19BR2 and 18S‐Euka02 would detect most taxa, though adding 16S‐Inse01 would provide a more complete picture of community composition. To facilitate multimarker approaches, efforts are needed to greatly expand the existing barcode reference databases, particularly in less‐studied geographic regions. Using this and other refinements, it is likely that biases of metabarcoding from preservative ethanol will be considerably reduced, thereby facilitating the use of this approach in molecular biomonitoring (Elbrecht et al., 2017; Emilson et al., 2017; Serrana et al., 2019).

## AUTHOR CONTRIBUTIONS

All authors contributed to the research experiment, and they reviewed and approved the final manuscript. The research was designed by F.M.S.M., P.B., P.T. and M.J.F. The fieldwork and ethanol sampling were conducted by M.J.F. and S.R.Q.S., and the morphological identifications were performed by S.R.Q.S. The laboratory was conducted by F.M.S.M. and A.B. The in silico analyses were performed by BE. The bioinformatic analyses were conducted by F.M.S.M., and with P.B. and M.P. analysed the data. The manuscript was drafted by F.M.S.M. and P.B., with contributions from all coauthors.

## Supporting information

Supplementary Material

## Data Availability

Raw sequencing data are provided as FASTQ files in ENA project accession no. PRJEB39471, sample accession nos. ERS4834804‐ERS4834899. All data needed to replicate the analyses reported in this study (scripts and metadata) are available at the BioStudies repository (https://www.ebi.ac.uk/biostudies/studies/S‐BSST431).
