# Supplementary material for: Modelling technical and biological biases in macroinvertebrate community assessment from bulk preservative using multiple metabarcoding markers
Source: Mol Ecol. 2020 Oct 12;30(13):3221–38. doi: 10.1111/mec.15620 (PMC8359330; doi:10.1111/mec.15620)
Supplement: Supplementary file 1 — Supplementary Material [file MEC-30-3221-s001.pdf]

MOLECULAR ECOLOGY

Supplemental Information for:

Modelling technical and biological biases in macroinvertebrate community assessment from bulk preservative using multiple metabarcoding markers

Filipa M.S. Martins, Miguel Porto, Maria J. Feio, Bastian Egeter, Aurélie Bonin, Sónia R.Q. Serra, Pierre Taberlet, Pedro Beja

Table of Contents:

**Supplementary Tables**	Page 2
**Supplementary Figures**	Page 13

MOLECULAR ECOLOGY

Supplemental Tables

Table S1 Composition of the DNA mock sample used as positive control during library preparation. For each family, we provide the number of taxa included, and the amount and percentage of DNA in the sample.

Order	Family	taxa	DNA (ng)	% DNA	Order	Family	taxa	DNA (ng)	% DNA
**Coleoptera**	Chrysomelidae	1	0.014	0.1%	**Plecoptera**	Chloroperlidae	1	0.389	2.9%
	Dryopidae	1	0.016	0.1%		Nemouridae	1	0.037	0.3%
	Dytiscidae	2	0.052	0.4%		Perlidae	2	1.968	14.6%
	Gyrinidae	1	0.030	0.2%		Perlodidae	3	0.377	2.8%
	Haliplidae	1	0.023	0.2%	**Trichoptera**	Brachycentridae	2	0.179	1.3%
	Helophoridae	1	0.041	0.3%		Ecnomidae	1	0.021	0.2%
	Hydrochidae	1	0.032	0.2%		Glossosomatidae	3	0.244	1.8%
	Hydrophilidae	1	0.178	1.3%		Goeridae	1	0.244	1.8%
	Scirtidae	1	0.002	0.01%		Hydropsychidae	3	0.282	2.1%
**Diptera**	Anthomyiidae	1	0.023	0.2%		Hydroptilidae	2	0.059	0.4%
	Ceratopogonidae	1	0.037	0.3%		Lepidostomatidae	1	0.094	0.7%
	Dolichopodidae	1	0.359	2.7%		Leptoceridae	5	0.325	2.4%
	Empididae	1	0.030	0.2%		Limnephilidae	2	0.142	1.1%
	Rhagionidae	1	1.502	11.2%		Odontoceridae	1	0.121	0.9%
	Sciomyzidae	1	0.217	1.6%		Philopotamidae	3	0.302	2.2%
	Simuliidae	1	0.053	0.4%		Polycentropodidae	2	0.155	1.2%
	Stratiomyidae	1	0.766	5.7%		Psychomyiidae	3	0.122	0.9%
	Syrphidae	1	0.325	2.4%		Rhyacophilidae	2	0.194	1.4%
	Tabanidae	1	0.407	3.0%		Sericostomatidae	1	0.122	0.9%
**Hemiptera**	Corixidae	1	0.037	0.3%		Uenoidae	2	0.225	1.7%
	Gerridae	2	0.302	2.2%	**Odonata**	Aeshnidae	1	0.089	0.7%
	Hydrometridae	1	0.041	0.3%		Calopterygidae	1	0.105	0.8%
	Nepidae	1	0.055	0.4%		Coenagrionidae	1	0.123	0.9%
	Notonectidae	1	0.563	4.2%		Gomphidae	1	0.279	2.1%
**Megaloptera**	Sialidae	1	0.238	1.8%		Lestidae	1	0.037	0.3%
**Ephemeroptera**	Baetidae	1	0.053	0.4%		Libellulidae	2	0.608	4.5%
	Heptageniidae	1	1.065	7.9%		Platycnemididae	1	0.123	0.9%
	Leptophlebiidae	1	0.037	0.3%					

MOLECULAR ECOLOGY

Table S2 Total number of individuals and total number of families recorded in each of 80 benthic macroinvertebrate samples collected at river sites in Central Portugal, under a Water Framework Directive sampling program. Estimates were based on morphotaxonomic identification at family level (except Oligochaeta and Hydracarina)

SampleID	#Individuals	#Families	SampleID	#Individuals	#Families
Morpho_EtOH0248	927	18	Morpho_EtOH0288	1316	36
Morpho_EtOH0249	1302	25	Morpho_EtOH0289	1753	36
Morpho_EtOH0250	737	23	Morpho_EtOH0290	1250	36
Morpho_EtOH0251	439	17	Morpho_EtOH0291	633	32
Morpho_EtOH0252	1091	21	Morpho_EtOH0292	830	31
Morpho_EtOH0253	524	24	Morpho_EtOH0293	1650	31
Morpho_EtOH0254	1248	22	Morpho_EtOH0294	981	28
Morpho_EtOH0255	446	33	Morpho_EtOH0295	238	17
Morpho_EtOH0256	2583	22	Morpho_EtOH0296	800	16
Morpho_EtOH0257	375	19	Morpho_EtOH0297	3131	29
Morpho_EtOH0258	2674	26	Morpho_EtOH0298	936	29
Morpho_EtOH0259	1063	33	Morpho_EtOH0299	1338	32
Morpho_EtOH0260	2270	28	Morpho_EtOH0300	352	29
Morpho_EtOH0261	1091	19	Morpho_EtOH0301	7908	36
Morpho_EtOH0262	845	23	Morpho_EtOH0302	1063	28
Morpho_EtOH0263	1027	24	Morpho_EtOH0303	1235	35
Morpho_EtOH0264	248	20	Morpho_EtOH0304	854	25
Morpho_EtOH0265	1323	15	Morpho_EtOH0305	891	31
Morpho_EtOH0266	164	18	Morpho_EtOH0306	870	33
Morpho_EtOH0267	258	20	Morpho_EtOH0307	550	36
Morpho_EtOH0268	201	14	Morpho_EtOH0308	912	35
Morpho_EtOH0269	797	21	Morpho_EtOH0309	637	33
Morpho_EtOH0270	177	13	Morpho_EtOH0310	790	35
Morpho_EtOH0271	1187	17	Morpho_EtOH0311	499	34
Morpho_EtOH0272	236	12	Morpho_EtOH0312	1225	39
Morpho_EtOH0273	475	11	Morpho_EtOH0313	1211	33
Morpho_EtOH0274	297	10	Morpho_EtOH0314	812	32
Morpho_EtOH0275	262	13	Morpho_EtOH0315	579	40
Morpho_EtOH0276	288	10	Morpho_EtOH0316	554	31
Morpho_EtOH0277	237	8	Morpho_EtOH0317	2022	41
Morpho_EtOH0278	264	9	Morpho_EtOH0318	1179	34
Morpho_EtOH0279	128	8	Morpho_EtOH0319	745	28
Morpho_EtOH0280	87	9	Morpho_EtOH0320	1179	40
Morpho_EtOH0281	132	12	Morpho_EtOH0321	968	30
Morpho_EtOH0282	81	4	Morpho_EtOH0322	623	22
Morpho_EtOH0283	130	5	Morpho_EtOH0323	106	14
Morpho_EtOH0284	256	14	Morpho_EtOH0324	1775	18
Morpho_EtOH0285	1198	26	Morpho_EtOH0325	1017	20
Morpho_EtOH0286	997	23	Morpho_EtOH0326	403	16
Morpho_EtOH0287	400	23	Morpho_EtOH0327	55	7

MOLECULAR ECOLOGY

Table S3 Taxonomic composition of benthic macroinvertebrate communities estimated from the morphotaxonomic identification at family level (except Oligochaeta and Hydracarina) of samples collected at 80 river sites in Central Portugal. For each family, we indicate the number of sites where each family was detected (#Sites), the total number of individuals per family (#Ind), the mean (± standard deviation) and range (minimum-maximum) of individuals counted per site (#Ind per Site), and the body armouring categorisation used in data analysis.

Family name	#Sites	#Ind	#Ind per Site		Body armouring
			Mean ± SD	Minimum-Maximum	
**Aeshnidae**	30	111	3.70 ± 3.30	(1 – 13)	hard
**Ancylidae**	41	422	10.29 ± 11.54	(1 – 44)	cased
**Aphelocheiridae**	8	39	4.88 ± 6.13	(1 – 17)	hard
**Asellidae**	14	127	9.07 ± 14.13	(1 – 50)	hard
**Athericidae**	37	171	4.62 ± 4.73	(1 – 18)	soft
**Atyidae**	18	437	24.28 ± 33.84	(1 – 126)	hard
**Baetidae**	75	6923	92.31 ± 112.73	(1 – 654)	soft
**Beraeidae**	6	175	29.17 ± 60.77	(1 – 153)	cased
**Blephariceridae**	5	20	4.00 ± 3.94	(1 – 10)	cased
**Brachycentridae**	7	14	2.00 ± 1.91	(1 – 6)	cased
**Caenidae**	55	1346	24.47 ± 50.62	(1 – 339)	soft
**Calamoceratidae**	8	21	2.63 ± 1.69	(1 – 5)	cased
**Calopterygidae**	32	122	3.81 ± 4.25	(1 – 23)	soft
**Cambaridae**	14	45	3.21 ± 3.56	(1 – 12)	hard
**Capniidae**	2	22	11.00 ± 14.14	(1 – 21)	soft
**Ceratopogonidae**	52	265	5.10 ± 5.74	(1 – 33)	soft
**Chironomidae**	80	13070	163.38 ± 208.35	(5 – 1278)	soft
**Chloroperlidae**	16	313	19.56 ± 27.14	(1 – 85)	soft
**Chrysomelidae**	2	5	2.50 ± 0.71	(2 – 3)	hard
**Coenagrionidae**	1	4	4.00 ± 0.00	(4 – 4)	soft
**Corbiculidae**	11	109	9.91 ± 10.47	(2 – 38)	cased
**Cordulegastridae**	20	56	2.80 ± 2.46	(1 – 9)	hard
**Corixidae**	4	6	1.50 ± 0.58	(1 – 2)	hard
**Corophiidae**	1	4	4.00 ± 0.00	(4 – 4)	hard
**Dixidae**	19	66	3.47 ± 6.45	(1 – 24)	hard
**Dolichopodidae**	1	1	1.00 ± 0.00	(1 – 1)	soft
**Dryopidae**	20	22	1.10 ± 0.31	(1 – 2)	cased
**Dugesiidae**	26	219	8.42 ± 15.60	(1 – 62)	soft
**Dytiscidae**	33	203	6.15 ± 10.39	(1 – 52)	hard
**Elmidae**	64	3957	61.83 ± 100.81	(1 – 529)	hard
**Empididae**	35	146	4.17 ± 4.95	(1 – 21)	soft
**Ephemerellidae**	60	7931	132.18 ± 185.68	(1 – 932)	soft
**Ephemeridae**	13	60	4.62 ± 4.82	(1 – 15)	soft
**Erpobdellidae**	25	83	3.32 ± 5.51	(1 – 27)	soft
**Gammaridae**	12	3128	260.67 ± 432.27	(1 – 1117)	hard
**Gerridae**	15	30	2.00 ± 1.36	(1 – 5)	soft
**Glossiphoniidae**	7	9	1.29 ± 0.49	(1 – 2)	soft
**Glossosomatidae**	17	171	10.06 ± 15.72	(1 – 55)	cased
**Goeridae**	2	7	3.50 ± 3.54	(1 – 6)	cased
**Gomphidae**	39	520	13.33 ± 20.61	(1 – 98)	hard
**Gyrinidae**	26	91	3.50 ± 3.42	(1 – 14)	hard
**Haliplidae**	1	3	3.00 ± 0.00	(3 – 3)	hard
**Helicopsychidae**	1	1	1.00 ± 0.00	(1 – 1)	cased
**Helophoridae**	15	46	3.07 ± 4.06	(1 – 13)	hard

Family name	#Sites	#Ind	#Ind per Site		Body armouring
			Mean ± SD	Minimum-Maximum	
**Heptageniidae**	36	994	27.61 ± 33.92	(1 – 139)	soft
**Hydracarina**	15	34	2.27 ± 2.02	(1 – 9)	hard
**Hydraenidae**	33	266	8.06 ± 13.80	(1 – 73)	hard
**Hydrobiidae**	42	13166	313.48 ± 1161.95	(1 – 7163)	cased
**Hydrochidae**	1	1	1.00 ± 0.00	(1 – 1)	hard
**Hydrometridae**	6	8	1.33 ± 0.82	(1 – 3)	hard
**Hydrophilidae**	25	83	3.32 ± 3.45	(1 – 13)	hard
**Hydropsychidae**	62	1443	23.27 ± 38.89	(1 – 190)	soft
**Hydroptilidae**	24	251	10.46 ± 20.03	(1 – 88)	hard
**Lepidostomatidae**	17	97	5.71 ± 7.20	(1 – 25)	soft
**Leptoceridae**	31	257	8.29 ± 17.89	(1 – 100)	hard
**Leptophlebiidae**	46	1173	25.50 ± 39.22	(1 – 218)	soft
**Leuctridae**	33	685	20.76 ± 29.12	(1 – 119)	soft
**Libellulidae**	3	8	2.67 ± 2.08	(1 – 5)	hard
**Limnephilidae**	29	973	33.55 ± 59.05	(1 – 246)	cased
**Limoniidae**	33	257	7.79 ± 16.86	(1 – 89)	soft
**Lymnaeidae**	8	67	8.38 ± 11.44	(1 – 29)	cased
**Muscidae**	5	7	1.40 ± 0.55	(1 – 2)	soft
**Nemouridae**	16	189	11.81 ± 13.25	(1 – 52)	soft
**Nepidae**	1	1	1.00 ± 0.00	(1 – 1)	hard
**Neritidae**	1	187	187.00 ± 0.00	(187 – 187)	cased
**Noteridae**	3	3	1.00 ± 0.00	(1 – 1)	hard
**Notonectidae**	2	2	1.00 ± 0.00	(1 – 1)	hard
**Odontoceridae**	1	2	2.00 ± 0.00	(2 – 2)	cased
**Oligochaeta**	73	975	13.36 ± 27.65	(1 – 214)	soft
**Oligoneuriidae**	11	491	44.64 ± 79.11	(1 – 215)	soft
**Perlidae**	6	117	19.50 ± 22.35	(1 – 50)	hard
**Perlodidae**	25	759	30.36 ± 27.30	(1 – 93)	hard
**Philopotamidae**	23	486	21.13 ± 48.13	(1 – 221)	soft
**Physidae**	27	527	19.52 ± 37.71	(1 – 170)	cased
**Planariidae**	14	58	4.14 ± 5.60	(1 – 22)	soft
**Planorbidae**	9	46	5.11 ± 5.90	(1 – 16)	cased
**Platycnemididae**	1	3	3.00 ± 0.00	(3 – 3)	hard
**Polycentropodidae**	23	180	7.83 ± 11.71	(1 – 42)	soft
**Psychodidae**	6	21	3.50 ± 5.21	(1 – 14)	hard
**Psychomyiidae**	18	48	2.67 ± 2.43	(1 – 11)	soft
**Rhagionidae**	15	37	2.47 ± 1.68	(1 – 7)	soft
**Rhyacophilidae**	43	275	6.40 ± 4.99	(1 – 18)	hard
**Sciomyzidae**	1	1	1.00 ± 0.00	(1 – 1)	soft
**Scirtidae**	13	80	6.15 ± 5.81	(1 – 20)	hard
**Sericostomatidae**	14	72	5.14 ± 5.70	(1 – 24)	hard
**Sialidae**	1	1	1.00 ± 0.00	(1 – 1)	hard
**Simuliidae**	72	9072	126.00 ± 293.94	(1 – 2304)	soft
**Siphlonuridae**	6	114	19.00 ± 24.56	(2 – 64)	soft
**Sphaeriidae**	12	104	8.67 ± 15.51	(1 – 55)	cased
**Stratiomyiidae**	1	16	16.00 ± 0.00	(16 – 16)	soft
**Tabanidae**	8	24	3.00 ± 2.88	(1 – 8)	soft
**Tipulidae**	27	50	1.85 ± 1.29	(1 – 5)	soft
**Uenoidae**	4	86	21.50 ± 19.00	(1 – 43)	cased
**Veliidae**	4	17	4.25 ± 3.59	(1 – 9)	soft

MOLECULAR ECOLOGY

Table S4 Summary results of *in silico* and *in vitro* evaluations of marker adequacy for detecting benthic macroinvertebrate taxa potentially occurring in samples collected in rivers from Central Portugal. For each taxon, we provide: “barc” - number of barcode sequences that comprise the insert originated by the desired marker based only on the Genbank database; “bind” - number of those sequences that comprise the desired insert and the flanking regions for primer binding; “range” - minimum-maximum insert sizes recovered from sequences according to primer binding sites; “amp” - number of unique barcode sequences that were amplified by each primer set for a maximum number of mismatches of 5; “res” - percentage of barcodes that were identified up to family level. In marker adequacy, values in “in silico” and “in vitro” columns refer to detection (1) or non-detection (0) at the respective analysis, and “refDB” to whether barcodes with the desired insert are available (1) or not (0) in both public (Genbank and BOLD) and in-house databases (IBI-CIBIO and aquaDNA-LECA) for taxonomic assignment.

		COI-M19BR2								16S-Inse01								18S-Euka02							
							marker adequacy								marker adequacy								marker adequacy		
**Taxonomic Group**	**Family**	**barc**	**bind**	**range**	**amp**	**res**	***in silico***	***in vitro***	**refDB**	**barc**	**bind**	**range**	**amp**	**res**	***in silico***	***in vitro***	**refDB**	**barc**	**bind**	**range**	**amp**	**res**	***in silico***	***in vitro***	**refDB**
**Annelida**	**Oligochaeta**	1203	106	307-315	101	100	**1**		**1**	1372	1371	72-203	1356	98.14	**1**		**1**	633	633	76-103	625	64.27	**1**		**1**
	**Erpobdellidae**	93	8	313-313	8	100	**1**		**1**	3	3	125-127	3	66.67	**1**		**1**	17	17	111-112	17	100	**1**		**1**
	**Glossiphoniidae**	81	4	313-313	4	100	**1**		**1**	2	2	126-127	2	100	**1**		**1**	37	37	100-101	36	100	**1**		**1**
**Mollusca**	**Ancylidae**	29	5	313-313	5	100	**1**		**1**	125	125	97-108	124	98.57	**1**		**1**	2	2	101-101	2	100	**1**		**1**
	**Lymnaeidae**	192	2	313-313	2	100	**1**		**1**	80	80	97-109	80	100	**1**		**1**	17	17	75-106	17	100	**1**		**1**
	**Physidae**	33	2	313-313	2	100	**1**		**1**	34	34	113-332	31	100	**1**		**1**	4	4	101-101	4	0	**0**		**1**
	**Planorbidae**	206	36	313-313	36	100	**1**		**1**	91	91	83-112	90	100	**1**		**1**	31	31	101-102	31	100	**1**		**1**
	**Neritidae**	89	14	313-313	14	100	**1**		**1**	134	134	135-164	134	99.66	**1**		**1**	11	11	99-100	11	83.33	**1**		**1**
	**Hydrobiidae**	193	7	313-314	7	100	**1**		**1**	179	179	115-280	177	100	**1**		**1**	2	2	99-101	2	100	**1**		**1**
	**Corbiculidae**	56	5	313-313	5	100	**1**		**1**	87	86	114-124	85	100	**1**		**1**	3	3	101-101	3	66.67	**1**		**1**
	**Sphaeriidae**	3	0	-	0	0	**0**		**1**	96	96	102-121	96	100	**1**		**1**	6	6	101-101	6	100	**1**		**1**
**Platyhelminthes**	**Dugesiidae**	112	109	307-310	89	100	**1**		**1**	3	3	73-86	3	100	**1**		**1**	41	41	68-103	40	100	**1**		**1**
	**Planariidae**	3	3	310-310	3	100	**1**		**1**	1	1	76-76	1	100	**1**		**1**	7	7	99-100	6	100	**1**		**1**
**Malacostraca**	**Corophiidae**	15	1	313-313	1	100	**1**		**1**	1	1	145-145	1	100	**1**		**1**	2	2	249-308	0	0	**0**		**1**
	**Gammaridae**	265	13	313-314	13	100	**1**		**1**	410	336	112-162	330	100	**1**		**1**	203	203	199-332	1	100	**1**		**1**

MOLECULAR ECOLOGY

Taxonomic Group	Family	COI-M19BR2								16S-Inse01								18S-Euka02							
							marker adequacy								marker adequacy								marker adequacy		
		barc	bind	range	amp	res	*in silico*	*in vitro*	refDB	barc	bind	range	amp	res	*in silico*	*in vitro*	refDB	barc	bind	range	amp	res	*in silico*	*in vitro*	refDB
	Atyidae	60	16	313-315	16	100	1		1	325	323	114-169	321	100	1		1	28	28	107-115	28	92.31	1		1
Malacostraca	Cambaridae	261	142	313-313	132	100	1		1	244	244	149-157	237	100	1		1	30	30	113-113	30	0	0		1
	Asellidae	64	0	-	0	0	0		1	127	127	111-139	119	100	1		1	4	4	220-221	0	0	0		1
Trombidiformes	Hydracarina	758	9	313-313	9	100	1		1	0	0	-	0	0	0		0	60	60	99-163	60	100	1		1
Coleoptera	Chrysomelidae	2442	471	312-314	434	99.76	1	1	1	1036	1031	137-166	1006	99.91	1	0	1	666	648	142-186	632	99.75	1	0	1
	Dryopidae	4	0	-	0	0	0	0	1	33	33	153-158	32	96.97	1	0	1	22	22	154-158	22	100	1	1	1
	Dytiscidae	543	180	313	170	100	1	1	1	1258	1258	149-164	1232	100	1	0	1	99	99	150-231	2	100	1	1	1
	Elmidae	70	0	-	0	0	0		1	62	62	154-160	62	100	1		1	49	49	157-159	49	100	1		1
	Gyrinidae	122	88	313-313	88	100	1	1	1	20	20	155-159	20	100	1	0	1	6	6	191-259	2	100	1	0	1
	Haliplidae	10	1	313-313	1	100	1	0	1	6	6	157-160	6	100	1	0	1	6	6	215-267	0	0	0	0	1
	Helophoridae	30	4	313-313	4	100	1	1	1	5	5	157-159	5	100	1	0	1	6	6	158-174	6	66.67	1	1	1
	Hydraenidae	324	3	313-313	3	100	1		1	217	216	145-157	216	100	1		1	10	10	156-162	10	100	1		1
	Hydrochidae	14	0	-	0	0	0	1	1	3	3	155-156	3	100	1	0	1	4	4	158-158	4	75	1	0	1
	Hydrophilidae	228	30	313-313	30	100	1	1	1	163	161	151-161	161	99.42	1	1	1	287	287	155-174	285	98.76	1	1	1
	Noteridae	4	2	313-313	2	100	1		1	63	63	151-161	60	100	1		1	47	47	41-295	1	1	1		1
	Scirtidae	92	34	313-313	30	100	1	1	1	18	18	151-160	18	100	1	0	1	16	16	146-244	13	100	1	0	1
Diptera	Anthomyiidae	1453	21	313	21	95	1	1	1	13	12	156-158	11	90.91	1	1	1	1	1	204	0	0	0	0	1
	Athericidae	2	0	-	0	0	0		1	1	1	158-158	1	100	1		1	0	0	-	0	0	0		0
	Blephariceridae	6	1	313-313	1	100	1		1	2	2	156-156	2	100	1		1	1	1	225-225	0	0	0		1
	Ceratopogonidae	1441	112	310-313	111	100	1	0	1	58	51	154-166	50	100	1	0	1	6	6	184-186	5	100	1	0	1
	Chironomidae	6623	72	312-313	64	100	1		1	65	65	153-200	63	100	1		1	8	8	188-216	4	100	1		1
	Dixidae	37	0	-	0	0	0		1	3	3	156-156	3	100	1		1	2	2	190-190	2	100	1		1
	Dolichopodidae	777	2	313-313	2	100	1	1	1	385	385	152-169	370	100	1	0	1	75	75	201-204	13	100	1	0	1
	Empididae	713	35	313-313	35	100	1	1	1	12	12	156-160	12	100	1	1	1	8	8	205-207	0	0	0	0	1

MOLECULAR ECOLOGY

		COI-M19BR2								16S-Inse01								18S-Euka02							
							marker adequacy								marker adequacy								marker adequacy		
**Taxonomic Group**	**Family**	barc	bind	range	amp	res	*in silico*	*in vitro*	refDB	barc	bind	range	amp	res	*in silico*	*in vitro*	refDB	barc	bind	range	amp	res	*in silico*	*in vitro*	refDB
**Diptera**	**Limoniidae**	304	0	-	0	0	**0**		**1**	18	18	156-158	18	100	**1**		**1**	1	1	212-212	0	0	**0**		**1**
	**Muscidae**	1276	82	313-320	82	98.25	**1**		**1**	118	118	150-160	118	96.83	**1**		**1**	3	3	186-204	1	100	**1**		**1**
	**Psychodidae**	450	58	313-313	55	100	**1**		**1**	47	18	156-161	18	100	**1**		**1**	86	86	164-316	51	100	**1**		**1**
	**Rhagionidae**	102	0	-	0	0	**0**	**1**	**1**	1	1	155-155	1	100	**1**	**0**	**1**	0	0	-	0	0	**0**		**0**
	**Sciomyzidae**	189	39	313-313	12	100	**1**	**0**	**1**	100	100	153-158	100	100	**1**	**0**	**1**	0	0	-	0	0	**0**		**0**
	**Simuliidae**	452	57	313-316	57	100	**1**	**1**	**1**	193	192	155-158	191	100	**1**	**1**	**1**	68	38	181-191	37	100	**1**	**0**	**1**
	**Stratiomyiidae**	130	3	313-313	3	100	**1**	**1**	**1**	33	33	155-158	33	100	**1**	**0**	**1**	1	1	207-207	0	0	**0**	**0**	**1**
	**Syrphidae**	1499	433	310-313	381	100	**1**	**1**	**1**	27	27	155-159	27	100	**1**	**0**	**1**	1	1	204	0	0	**0**	**0**	**1**
	**Tabanidae**	358	104	313-313	103	100	**1**	**1**	**1**	12	12	156-157	12	100	**1**	**1**	**1**	3	3	202-208	1	100	**1**	**0**	**1**
	**Tipulidae**	194	1	313-313	1	100	**1**		**1**	3	3	156-157	3	100	**1**		**1**	20	19	213-242	0	0	**0**		**1**
**Hemiptera**	**Aphelocheiridae**	0	0	-	0	0	**0**		**1**	4	4	159-163	4	100	**1**		**1**	1	1	108-108	1	100	**1**		**1**
	**Corixidae**	25	2	313-314	2	100	**1**	**1**	**1**	10	10	156-162	10	100	**1**	**0**	**1**	4	4	107-109	4	75	**1**	**1**	**1**
	**Gerridae**	45	7	312-313	7	100	**1**	**1**	**1**	148	145	156-163	145	100	**1**	**1**	**1**	10	10	107-107	10	85.71	**1**	**1**	**1**
	**Hydrometridae**	5	1	313-313	1	100	**1**	**1**	**1**	7	7	158-161	7	100	**1**	**1**	**1**	1	1	108-108	1	100	**1**	**1**	**1**
	**Nepidae**	8	1	313-313	1	100	**1**	**1**	**1**	20	20	155-162	20	100	**1**	**1**	**1**	2	2	109-116	2	66.67	**1**	**1**	**1**
	**Notonectidae**	26	0	-	0	0	**0**	**1**	**1**	16	16	157-164	16	100	**1**	**0**	**1**	2	2	109-109	2	100	**1**	**1**	**1**
	**Veliidae**	24	9	313-313	9	100	**1**		**1**	71	70	154-161	69	100	**1**		**1**	7	7	107-205	6	100	**1**		**1**
**Megaloptera**	**Sialidae**	8	1	313-313	1	100	**1**	**1**	**1**	7	7	146-159	7	100	**1**	**1**	**1**	7	7	97-318	1	100	**1**	**0**	**1**
**Ephemeroptera**	**Baetidae**	313	27	311-313	27	100	**1**	**1**	**1**	94	94	140-155	93	100	**1**	**0**	**1**	71	70	104-105	69	100	**1**	**1**	**1**
	**Caenidae**	29	0	-	0	0	**0**		**1**	5	5	150-152	5	100	**1**		**1**	9	9	104-106	9	100	**1**		**1**
	**Ephemerellidae**	101	4	313-313	4	100	**1**		**1**	27	26	149-153	26	100	**1**		**1**	32	32	104-111	31	100	**1**		**1**
	**Ephemeridae**	12	1	313-313	1	100	**1**		**1**	8	8	151-154	8	100	**1**		**1**	8	8	104-104	7	66.67	**1**		**1**
	**Heptageniidae**	206	5	313-313	5	100	**1**	**1**	**1**	127	127	154-159	126	100	**1**	**1**	**1**	15	15	105-107	15	100	**1**	**1**	**1**

Taxonomic Group	Family	COI-M19BR2								16S-Inse01								18S-Euka02							
							marker adequacy								marker adequacy								marker adequacy		
		barc	bind	range	amp	res	*in silico*	*in vitro*	refDB	barc	bind	range	amp	res	*in silico*	*in vitro*	refDB	barc	bind	range	amp	res	*in silico*	*in vitro*	refDB
Ephemeroptera	Leptophlebiidae	134	40	313-313	40	100	**1**	**1**	**1**	9	9	148-151	9	100	**1**	**1**	**1**	14	14	105-106	14	80	**1**	**1**	**1**
	Oligoneuriidae	3	0	-	0	0	**0**		**1**	17	17	140-152	17	100	**1**		**1**	5	5	105-106	5	100	**1**		**1**
	Siphlonuridae	29	1	313-313	1	100	**1**		**1**	6	5	152-155	5	100	**1**		**1**	4	4	105-105	4	100	**1**		**1**
Plecoptera	Capniidae	25	6	313-313	6	100	**1**		**1**	7	7	156-157	7	100	**1**		**1**	12	12	126-126	12	100	**1**		**1**
	Chloroperlidae	35	5	313-313	5	100	**1**	**1**	**1**	12	12	154-159	12	100	**1**	**1**	**1**	12	12	122-124	12	71.43	**1**	**1**	**1**
	Leuctridae	59	10	313-313	10	100	**1**		**1**	11	11	156-159	11	100	**1**		**1**	11	11	119-126	11	100	**1**		**1**
	Nemouridae	104	5	313-313	5	100	**1**	**1**	**1**	27	27	156-158	25	100	**1**	**1**	**1**	19	19	115-121	19	100	**1**	**1**	**1**
	Perlidae	60	7	313-313	7	100	**1**	**1**	**1**	21	21	156-168	21	100	**1**	**1**	**1**	18	18	120-122	18	66.67	**1**	**1**	**1**
	Perlodidae	83	7	313-313	6	100	**1**	**1**	**1**	42	42	156-158	42	100	**1**	**1**	**1**	30	30	122-122	29	80	**1**	**0**	**1**
Trichoptera	Beraeidae	9	0	-	0	0	**0**		**1**	1	1	147-147	1	100	**1**		**1**	0	0	-	0	0	**0**		**1**
	Brachycentridae	40	1	313-313	1	100	**1**	**0**	**1**	0	0	-	0	0	**0**	**0**	**1**	1	1	-	0	0	**0**	**0**	**1**
	Calamoceratidae	104	1	313-313	1	100	**1**		**1**	0	0	-	0	0	**0**		**1**	0	0	-	0	0	**0**		**0**
	Ecnomidae	158	0	-	0	0	**0**	**1**	**1**	0	0	-	0	0	**0**	**0**	**1**	1	1	106	1	100	**1**	**1**	**1**
	Glossosomatidae	211	0	-	0	0	**0**	**1**	**1**	0	0	-	0	0	**0**	**0**	**1**	1	1	188-188	1	100	**1**	**0**	**1**
	Goeridae	49	1	313-313	1	100	**1**	**1**	**1**	0	0	-	0	0	**0**	**0**	**1**	0	0	-	0	0	**0**	**0**	**1**
	Helicopsychidae	74	1	313-313	1	100	**1**		**1**	5	5	137-147	5	100	**1**		**1**	0	0	-	0	0	**0**		**0**
	Hydropsychidae	1224	19	313-313	19	100	**1**	**1**	**1**	11	11	146-197	9	100	**1**	**0**	**1**	10	10	135-175	9	100	**1**	**1**	**1**
	Hydroptilidae	313	2	313-313	2	100	**1**	**1**	**1**	0	0	-	0	0	**0**	**0**	**1**	2	2	165-174	2	100	**1**	**0**	**1**
	Lepidostomatidae	111	7	313-313	7	100	**1**	**1**	**1**	0	0	-	0	0	**0**	**0**	**1**	0	0	-	0	0	**0**	**0**	**1**
	Leptoceridae	652	3	313-313	3	100	**1**	**1**	**1**	16	16	137-158	16	100	**1**	**0**	**1**	6	6	178-184	6	100	**1**	**1**	**1**
	Limnephilidae	602	19	313-313	14	100	**1**	**1**	**1**	67	67	166-170	65	100	**1**	**1**	**1**	7	7	176-176	5	100	**1**	**1**	**1**
	Odontoceridae	72	3	313-313	3	100	**1**	**1**	**1**	0	0	-	0	0	**0**		**0**	0	0	-	0	0	**0**	**0**	**1**
	Philopotamidae	364	3	313-313	3	100	**1**	**1**	**1**	0	0	-	0	0	**0**	**0**	**1**	1	1	136-136	1	100	**1**	**0**	**1**

MOLECULAR ECOLOGY

Taxonomic Group	Family	COI-M19BR2								16S-Inse01								18S-Euka02							
							marker adequacy								marker adequacy								marker adequacy		
		barc	bind	range	amp	res	*in silico*	*in vitro*	refDB	barc	bind	range	amp	res	*in silico*	*in vitro*	refDB	barc	bind	range	amp	res	*in silico*	*in vitro*	refDB
**Trichoptera**	**Polycentropodidae**	256	1	313-313	1	100	**1**	**1**	**1**	0	0	-	0	0	**0**	**0**	**1**	2	2	105-180	2	50	**1**	**0**	**1**
	**Psychomyiidae**	65	0	-	0	0	**0**	**1**	**1**	0	0	-	0	0	**0**	**0**	**1**	0	0	-	0	0	**0**	**1**	**1**
	**Rhyacophilidae**	181	11	313-313	11	100	**1**	**1**	**1**	1	1	135-135	1	100	**1**	**1**	**1**	0	0	-	0	0	**0**	**1**	**1**
	**Sericostomatidae**	50	1	313-313	1	100	**1**	**1**	**1**	5	5	137-160	5	100	**1**	**1**	**1**	0	0	-	0	0	**0**	**0**	**1**
	**Uenoidae**	58	3	313-313	3	100	**1**	**1**	**1**	1	1	168-168	1	100	**1**	**0**	**1**	0	0	-	0	0	**0**	**1**	**1**
**Odonata**	**Aeshnidae**	74	13	312-313	13	100	**1**	**1**	**1**	53	50	150-161	50	98.65	**1**	**1**	**1**	13	13	106-107	13	66.67	**1**	**1**	**1**
	**Calopterygidae**	28	4	313-313	4	100	**1**	**1**	**1**	59	57	151-161	57	98.81	**1**	**1**	**1**	43	43	106-106	43	50	**1**	**0**	**1**
	**Coenagrionidae**	265	15	310-313	15	100	**1**	**1**	**1**	188	185	156-164	184	99.58	**1**	**1**	**1**	74	74	106-107	74	75	**1**	**0**	**1**
	**Cordulegastridae**	10	2	313-313	1	100	**1**		**1**	20	20	158-160	20	100	**1**		**1**	5	5	106-106	5	0	**0**		**1**
	**Gomphidae**	143	17	313-313	17	94.44	**1**	**1**	**1**	177	67	146-161	67	98.89	**1**	**1**	**1**	27	27	105-106	25	100	**1**	**1**	**1**
	**Lestidae**	28	5	313	5	100	**1**	**0**	**1**	30	29	116-159	28	100	**1**	**0**	**1**	19	19	150	19	0	**0**	**0**	**1**
	**Libellulidae**	270	80	312-314	78	100	**1**	**1**	**1**	275	259	99-158	247	99.72	**1**	**1**	**1**	34	34	104-107	33	78.57	**1**	**1**	**1**
	**Platycnemididae**	37	2	313-313	2	100	**1**	**1**	**1**	61	61	115-160	60	97.75	**1**	**1**	**1**	11	10	106-106	10	50	**1**	**0**	**1**

MOLECULAR ECOLOGY

Table S5 Summary statistics on sequencing coverage and data processing for each of three markers (COI-M19BR2, 16S-Inse01, 18S-Euka02), and for the combination of the three markers (multi-marker), used to amplify the DNA extracted from the preservative ethanol of 80 benthic macroinvertebrate samples collected in Central Portugal.

	COI-M19BR2		16S-Inse01		18S-Euka02		multi-marker	
**DATA PROCESSING**								
**Step**	**read counts**	**no. of sequences**	**read counts**	**no. of sequences**	**read counts**	**no. of sequences**		
Raw PE reads	25,232,044		50,185,936		39,544,628			
Merging PE reads	12,376,870		24,161,120		18,827,189			
Demultiplexing	11,980,079		21,769,698		15,430,470			
Dereplication	11,980,079	3,317,589	21,769,698	681,831	15,430,470	568,549		
Attribute filtering	7,150,053	419,841	12,966,129	152,402	14,071,108	130,727		
Clustering 99%	7,150,053	18,641	12,965,307	65,100	14,070,604	61,167		
LFN filtering	6,474,291	10,146	12,542,664	26,978	11,670,158	33,318		
**TAXONOMIC ASSIGNMENT**								
**Step**	**cluster counts**	**no. of taxa**	**cluster counts**	**no. of taxa**	**cluster counts**	**no. of taxa**	**cluster counts**	**no. of taxa**
OTU ≥92% identity	5,347,137	7,039	10,331,705	19,736	11,306,597	29,754		
Merged by family	4,667,500	89	7,282,933	30	6,129,904	70	18,080,337	109
Families ≥0.01%	4,666,828	82	7,282,739	28	6,128,900	68	18,078,467	102
Families detected by morphotaxonomy (out of 94)	4,534,495	55	7,025,168	24	5,576,808	40	17,136,471	62

MOLECULAR ECOLOGY

Table S6 Summary results of Generalised Linear Mixed Models (GLMMs) used to assess the relations between family-level detection probabilities and body armouring (hard [reference category]; cased, and soft bodied), absolute and relative abundances, taxa richness and per-sample sequencing depth to control for variation in sequencing read count among samples. Separate models were built for each of the three markers (COI-M19BR2, 16S-Inse01, 18S-Euka02), and for the combination of the three markers (multi-marker). For each variable in each model we provide the regression coefficient (Coef), the standard error of the coefficient estimate (SE), the *z*-value and the corresponding *P*-value (*P*).

	**COI-M19BR2**				**16S-Inse01**			
**Fixed effects**	**Coef**	**SE**	***z***	***P***	**Coef**	**SE**	***z***	***P***
Intercept	-3.074	0.522	-5.886	3.95E-09***	-5.865	0.913	-6.424	1.33E-10***
Soft-bodied	2.023	0.654	3.095	0.002**	2.000	1.021	1.958	0.050*ns*
Cased body	1.118	0.832	1.345	0.179*ns*	n.a.	n.a.	n.a.	n.a.
Absolute abundance	-0.101	0.089	-1.139	0.255*ns*	-0.335	0.147	-2.279	0.023*
Relative abundance	0.706	0.138	5.099	3.41E-07***	0.894	0.182	4.904	9.38E-07***
Richness	-0.125	0.106	-1.185	0.236*ns*	0.317	0.137	2.310	0.021*
Sequencing depth	0.626	0.109	5.759	8.47E-09***	0.496	0.137	3.631	2.83E-04***

	**18S-Euka02**				**multi-marker**			
**Fixed effects**	**Coef**	**SE**	***z***	***P***	**Coef**	**SE**	***z***	***P***
Intercept	-5.944	1.063	-5.59	2.27E-08***	-2.512	0.560	-4.485	7.28E-06***
Soft-bodied	3.141	1.223	2.569	0.010*	2.553	0.726	3.514	4.41E-04***
Cased body	3.017	1.546	1.951	0.051*ns*	1.806	0.912	1.980	0.048*
Absolute abundance	-0.02	0.301	-0.065	0.948*ns*	0.017	0.545	0.032	0.975*ns*
Relative abundance	0.681	0.197	3.463	5.35E-04***	1.131	0.285	3.971	7.16E-05***
Richness	0.246	0.141	1.748	0.08*ns*	0.072	0.113	0.638	0.523*ns*
Sequencing depth	0.285	0.141	2.025	0.043*	0.531	0.114	4.675	2.93E-06***

*****P*<0.001, ***P*<0.01, **P*<0.05, *ns P*>0.05.

MOLECULAR ECOLOGY

Supplemental Figures

	A	B	C	D	E	F	G	H
12	EtOH0321	EtOH0322	EtOH0323	MEXTneg1	EtOH0324	EtOH0325	EtOH0326	EtOH0327
11	EtOH0315	EtOH0316	MEXTneg2	EtOH0317	MPCRneg3	EtOH0318	EtOH0319	EtOH0320
10	EtOH0309	MEXTneg3	EtOH0310	EtOH0311	EtOH0312	MPCRneg2	EtOH0313	EtOH0314
9	MEXTneg4	EtOH0303	EtOH0304	EtOH0305	EtOH0306	EtOH0307	MPCRneg1	EtOH0308
8	EtOH0297	MPCRpos1	EtOH0298	EtOH0299	EtOH0300	EtOH0301	EtOH0302	MBlk8
7	EtOH0290	EtOH0291	EtOH0292	EtOH0293	EtOH0294	EtOH0295	MBlk7	EtOH0296
6	EtOH0283	EtOH0284	EtOH0285	EtOH0286	EtOH0287	MBlk6	EtOH0288	EtOH0289
5	EtOH0276	EtOH0277	EtOH0278	EtOH0279	MBlk5	EtOH0280	EtOH0281	EtOH0282
4	EtOH0269	EtOH0270	EtOH0271	MBlk4	EtOH0272	EtOH0273	EtOH0274	EtOH0275
3	EtOH0262	EtOH0263	MBlk3	EtOH0264	EtOH0265	EtOH0266	EtOH0267	EtOH0268
2	EtOH0255	MBlk2	EtOH0256	EtOH0257	EtOH0258	EtOH0259	EtOH0260	EtOH0261
1	MBlk1	EtOH0248	EtOH0249	EtOH0250	EtOH0251	EtOH0252	EtOH0253	EtOH0254

Legend	
(black)	**Tag/Index negative** control
(dark blue-grey)	**PCR negative** control
(orange)	**Extraction negative** control
(green)	**Positive** control
(light grey)	**Sample**

Figure S1 PCR plate layout.

MOLECULAR ECOLOGY

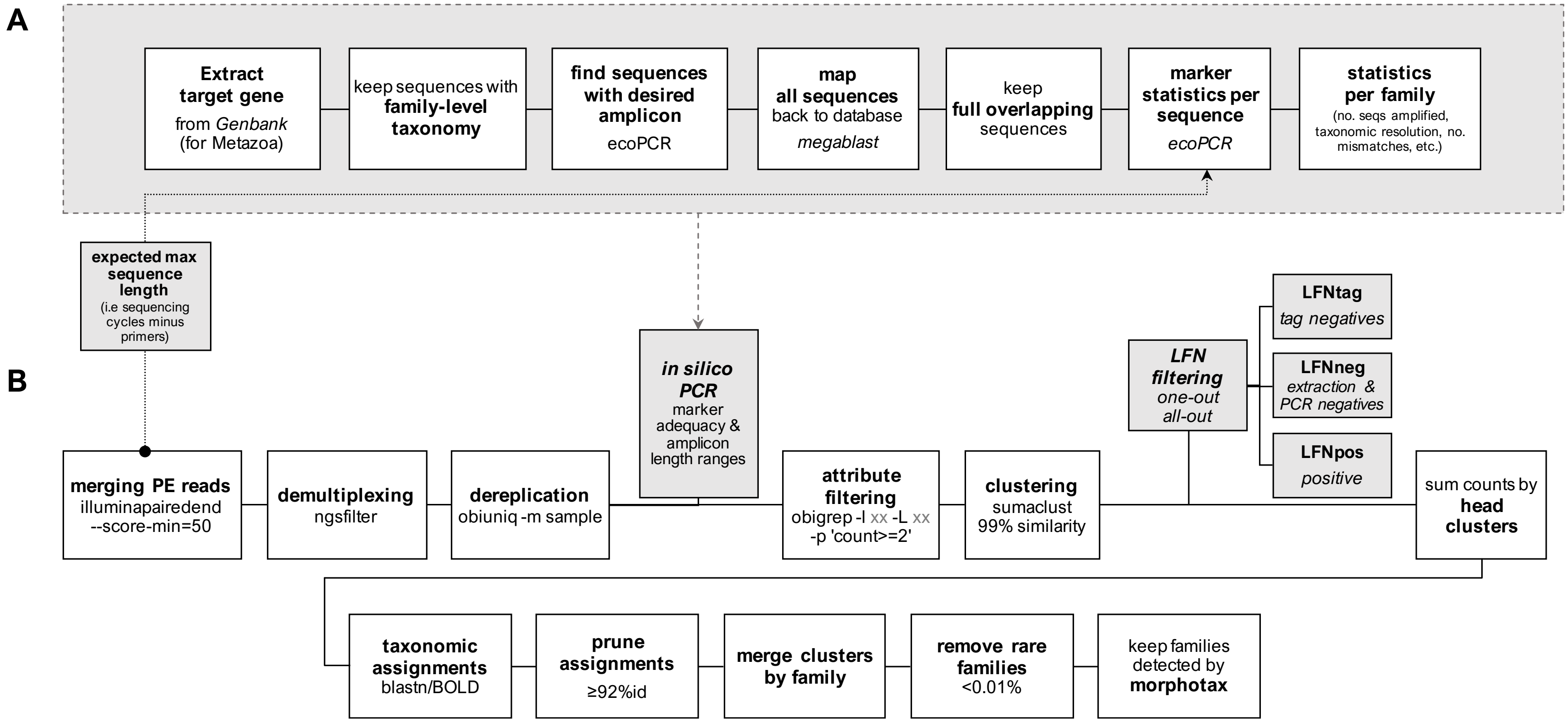

Figure S2 Scheme of the bioinformatic analysis used for (A) *in silico* testing and (B) processing of metabarcoding data.

MOLECULAR ECOLOGY

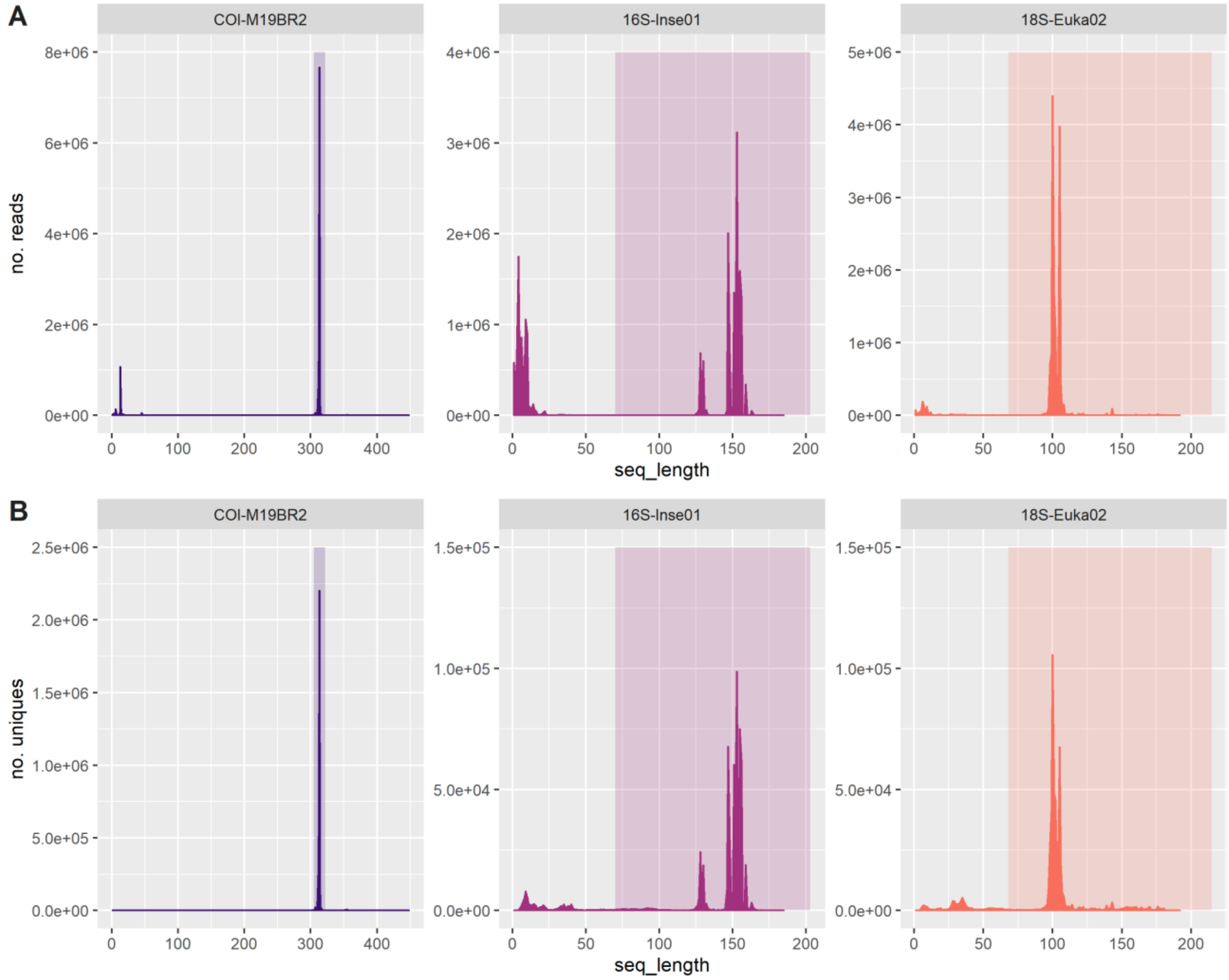

Figure S3 Distribution of (A) sequencing read counts and (B) unique sequences after dereplication (bars) overlaid by amplicon length ranges (shading) estimated *in silico* (Figure S2A) and used for filtering metabarcoding data (attribute filtering; Figure S2B), for each of the three DNA markers used in the study. Separate results are shown for each of three markers (COI-M19BR2, 16S-Inse01, 18S-Euka02) used in this study.

MOLECULAR ECOLOGY

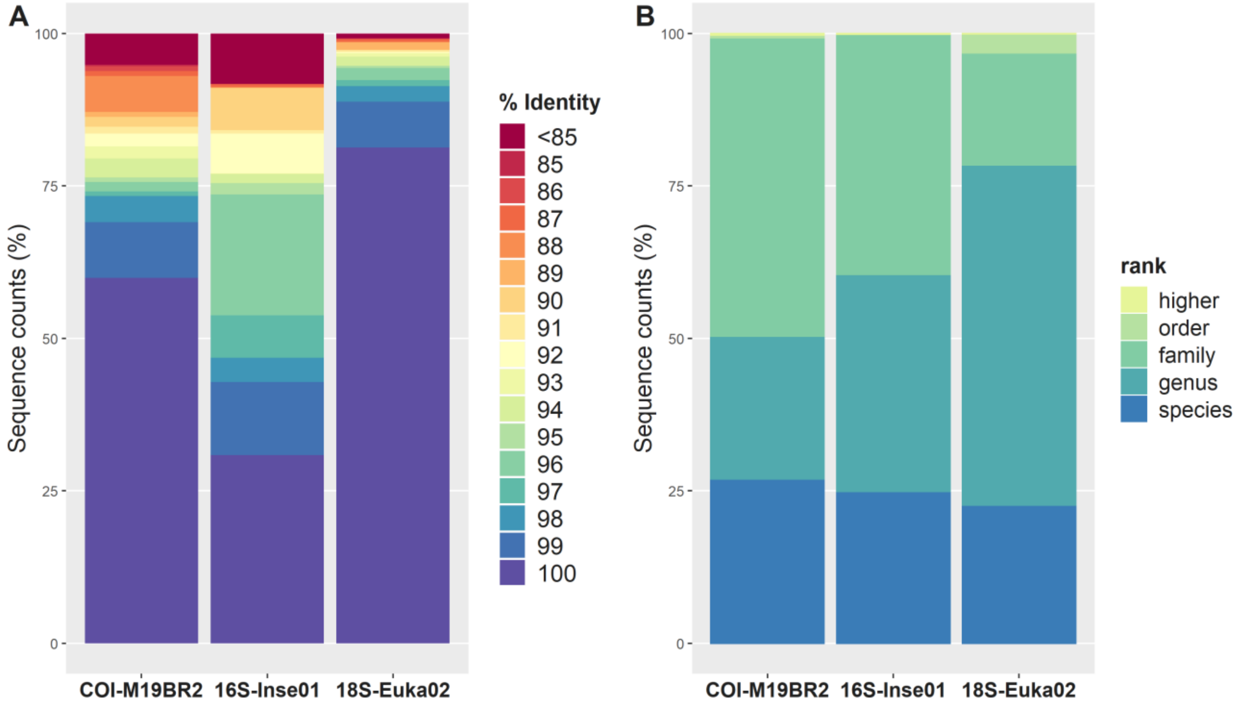

Figure S4 Percentage of reads (sequence counts) by (A) percentage of identity across cluster sequences and (B) taxonomic rank of clusters assigned to targeted macroinvertebrate taxa (≥92% identity). Separate results are shown for each of three markers (COI-M19BR2, 16S-Inse01, 18S-Euka02) used in this study.

MOLECULAR ECOLOGY

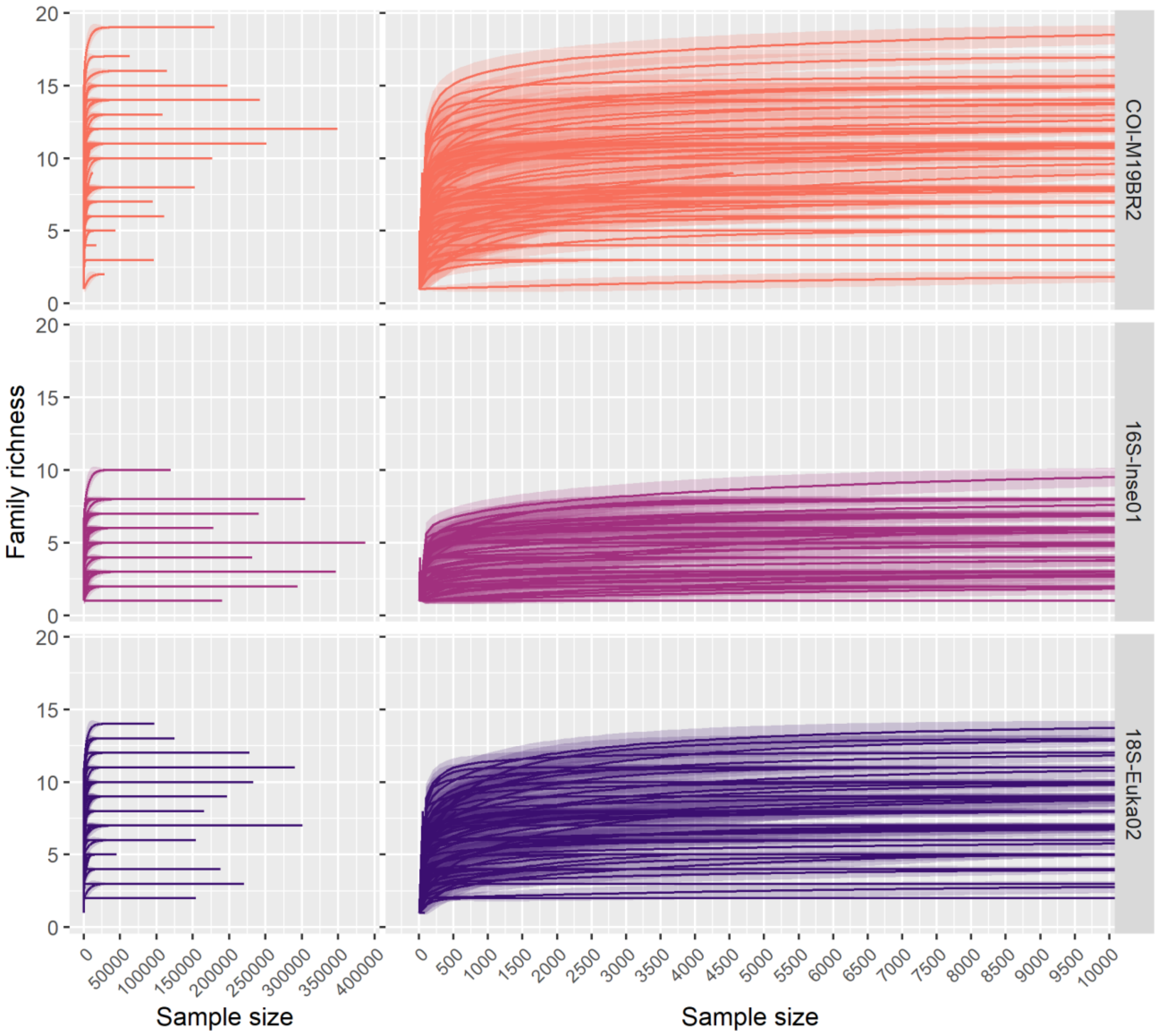

Figure S5 Rarefaction curves showing the accumulation of targeted macroinvertebrate families (Family richness) recovered by each of three individual markers (COI-M19BR2, 16S-Inse01, 18S-Euka02) in relation to read counts per sample (Sample size). Panels in the left represent rarefaction curves across the entire range of coverages obtained in our samples, while in the right panels we have zoomed in on read counts up to 10,000.

MOLECULAR ECOLOGY

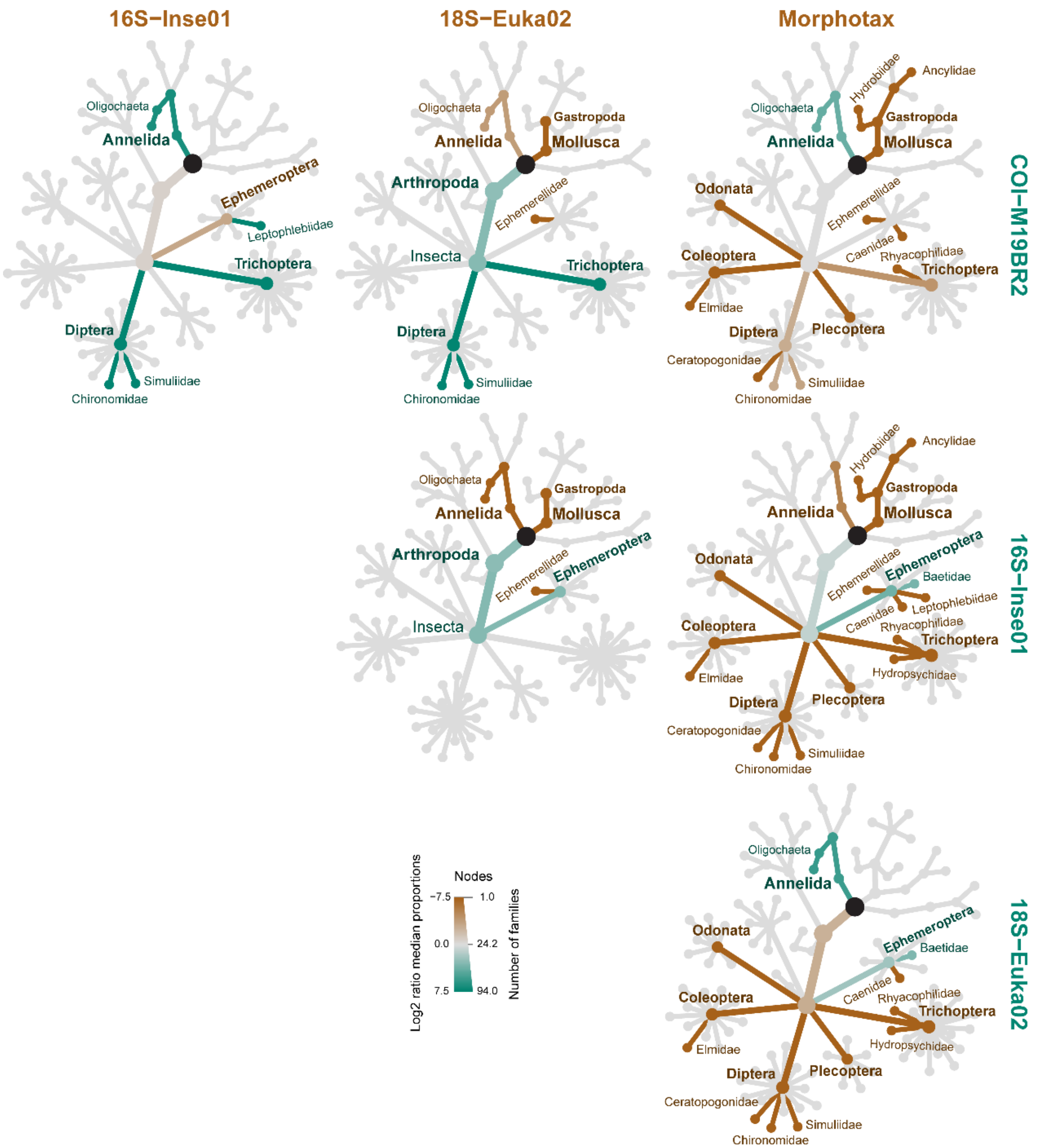

Figure S6 Pairwise differential heat trees showing significant differences in the taxonomic composition of macroinvertebrate communities retrieved using either morphotaxonomy or each of the three DNA markers used in this study. Heat trees were produced considering the "abundance" of taxa estimated with either morphotaxonomy (counts of individuals) or DNA markers (counts of reads). Only families detected by morphotaxonomy (sample-wise) were considered. Tree tips represent families and internal nodes the corresponding taxonomic path. Black nodes denote the Animalia root.

MOLECULAR ECOLOGY

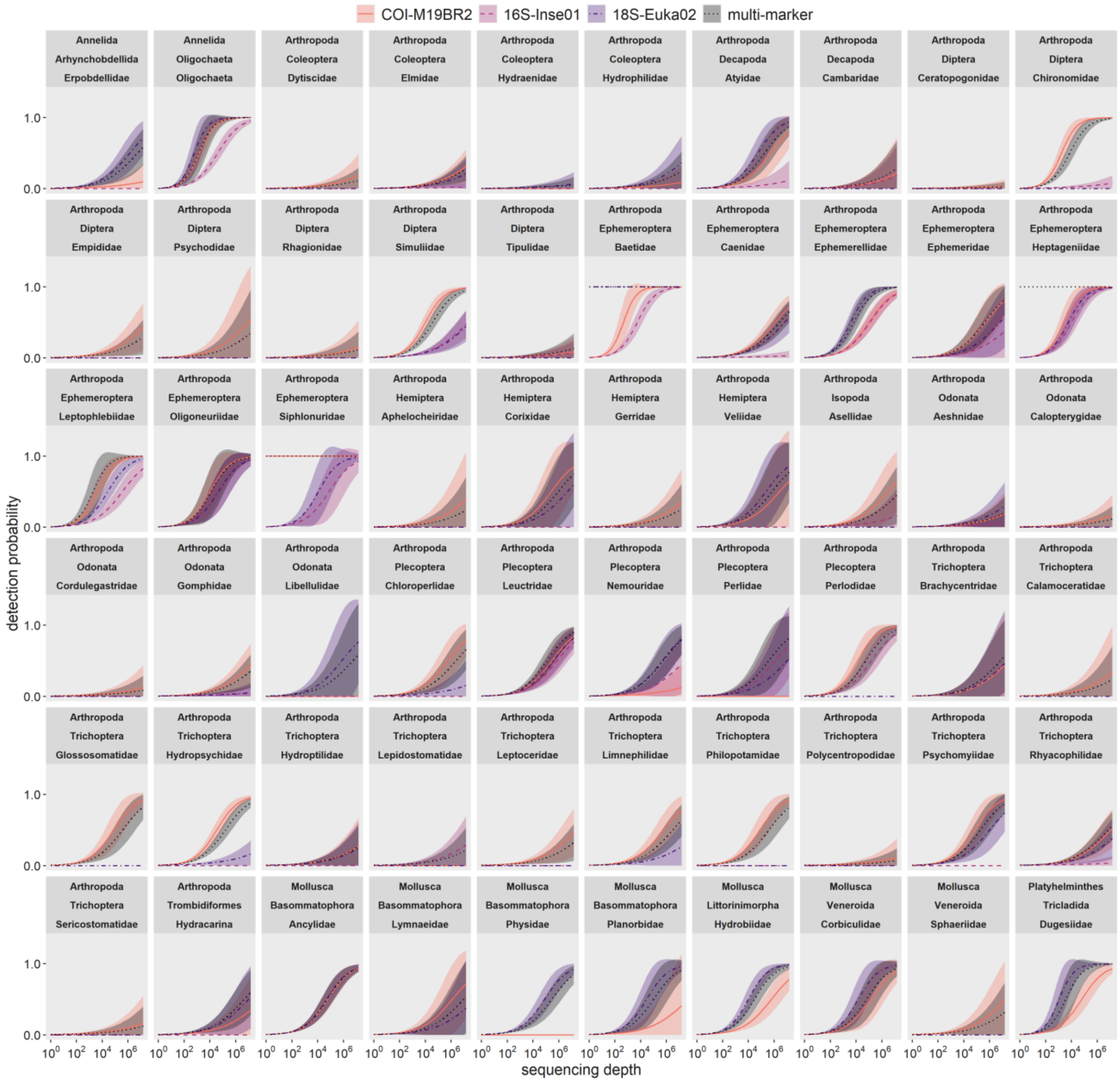

Figure S7 Detection probability curves (predicted values ± 95% confidence interval) showing variation in the probability of detecting through metabarcoding a macroinvertebrate taxon recorded in a sample by morphotaxonomy (i.e., true positives), as a function of per-sample sequencing depth. Curves were estimated using Generalized Linear Models with binomial distribution and logit link, with separate models fitted for each combination of macroinvertebrate taxa and each of three markers (COI-M19BR2, 16S-Inse01 or 18S-Euka02), or the multi-marker. Only taxa detected by morphotaxonomy (sample-wise) were considered.

MOLECULAR ECOLOGY

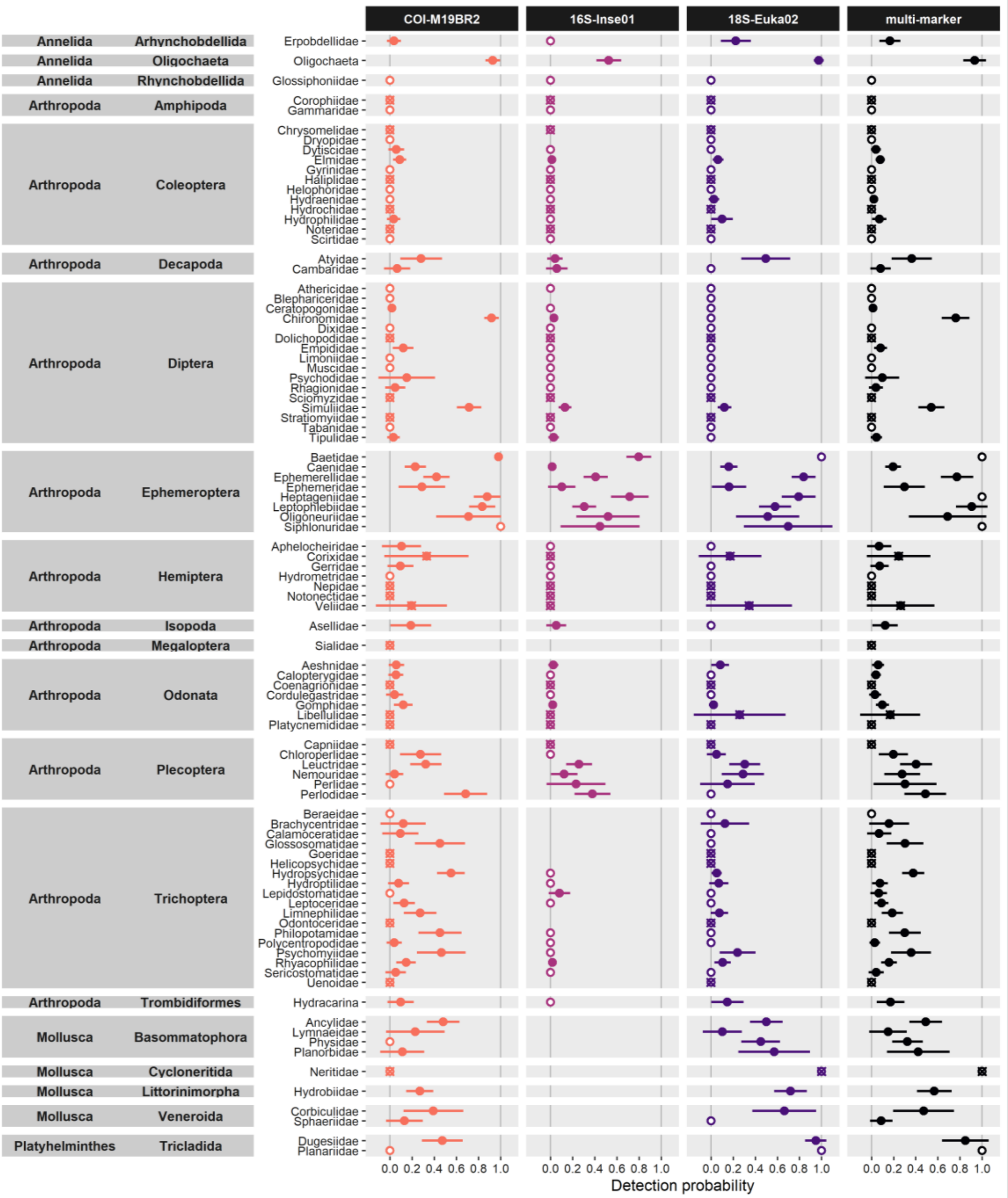

Figure S8 Detection probabilities (± 95% confidence intervals) of macroinvertebrate taxa using metabarcoding for a standard sequencing count of 50,000 reads, per each combination of macroinvertebrate taxon and each of three markers (COI-M19BR2, 16S-Inse01, 18S-Euka02), or the multi-marker. Estimates were made using the GLM shown in Figure S7. Only taxa detected by morphotaxonomy (sample-wise) were considered. White dots indicate taxa that were not detected by the respective marker or in which models failed to converge; crossed dots indicate rare families (<5 sites).
